# A comprehensive review of energy efficient routing protocols for query driven wireless sensor networks

**DOI:** 10.12688/f1000research.133874.1

**Published:** 2023-06-12

**Authors:** Punith Bekal, Pramod Kumar, Pallavi R Mane, Ghanshyam Prabhu

**Affiliations:** 1 Department of Electronics and Communication Engineering, Manipal Institute of Technology, Manipal Academy of Higher Education, MANIPAL, KARNATAKA, 576104, India; 2 Department of Information and Communication Technology, Manipal Institute of Technology, Manipal Academy of Higher Education, MANIPAL, KARNATAKA, 576104, India

**Keywords:** Energy efficient (EE), Internet of Things (IoT), Wireless sensor networks (WSNs)

## Abstract

In this current era of communications and networking, The Internet of things plays the main role in the making of smart communication and networking. In this article, we have focused on the literature survey on wireless sensor networks which are energy efficient. Various standard protocols are reviewed along with some enhanced protocols which makes the network energy efficient. The comparison of the standard and enhanced protocols with respect to various applications in wireless sensor networks is thoroughly done in this article. The outcomes of the enhanced protocols are also briefly discussed. For easier analysis to future researchers, a comparative table which lists the enhanced protocols which are compared with standard counterparts along with the factors for energy efficiency of the protocols. This article also comments on the issues and challenges of the protocols which can be further analyzed for making the wireless sensor network more energy efficient.

## Introduction

A wireless system is a type of data communication system that sends and receives data using radio frequency. It significantly decreased the requirement for wired connectivity. *Ad hoc* networks, on the other hand, operate without any infrastructure and hop between radio relays. ^1^ The distributed mode, or the base station (BS), will assist in coordinating the node for efficient data transmission. Despite the availability of a variety of information services, wireless sector networks (WSNs) guarantee accurate data by localizing in time and place in response to user demand. ^2^ A typical WSN functions better when it is under resource limitations like processor, bandwidth usage, storage capacity, *etc.* The sensor nodes (SNs) are randomly spread across the network area, efficiently managing the energy is a huge task. Due to vast developments taking place in wireless communication protocols, the sensor node internal components have become inexpensive, compact and more robust. A variety of services like routing, data processing, scheduling, key management, cryptography, *etc.* rely on sensor networks, which has attracted a lot of research attention. These services impose limitations on the network’s self-configurability, energy use, computing capacity, memory use, and other application-specific restrictions. ^3^

**Figure 1.**
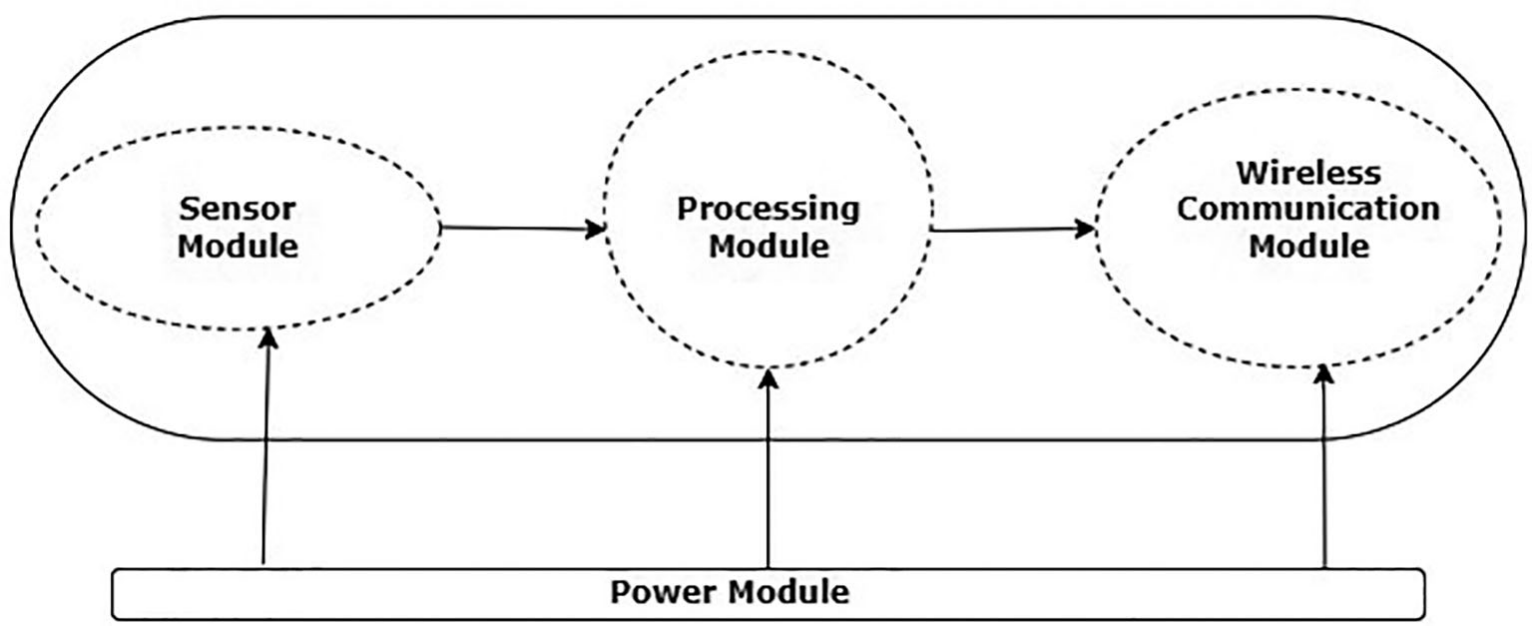
Wireless sensor network node.

SNs, which might be few or many in number, make up sensor networks. These nodes come in different sizes; depending on their size, the SNs function well in various domains. As a result of their special design, SNs in WSNs frequently include a microcontroller that controls the monitoring, a transceiver for creating radio waves, and various types of devices for wireless communication in addition to a source of energy like a battery. ^4^ The entire network functions simultaneously with sensors of different dimensions, and by using a routing mechanism, they are primarily concerned with getting the source data to the receiver node. ^4^ The WSNs basic architecture is depicted in Figure. 1.

The basic components of a WSN architecture are power supply module, wireless communication module, processing module, and sensor module. An analog-to-digital converter (ADC) and a sensor make up the sensor module. A sensor and an ADC are also included in the processing module. ^4^ The wireless module for communication has network layer and Mac layer protocols along with transceiver which helps in the data exchange between the nodes. The power supply module helps the nodes with the required energy for their operations. ^4^

**Figure 2.**
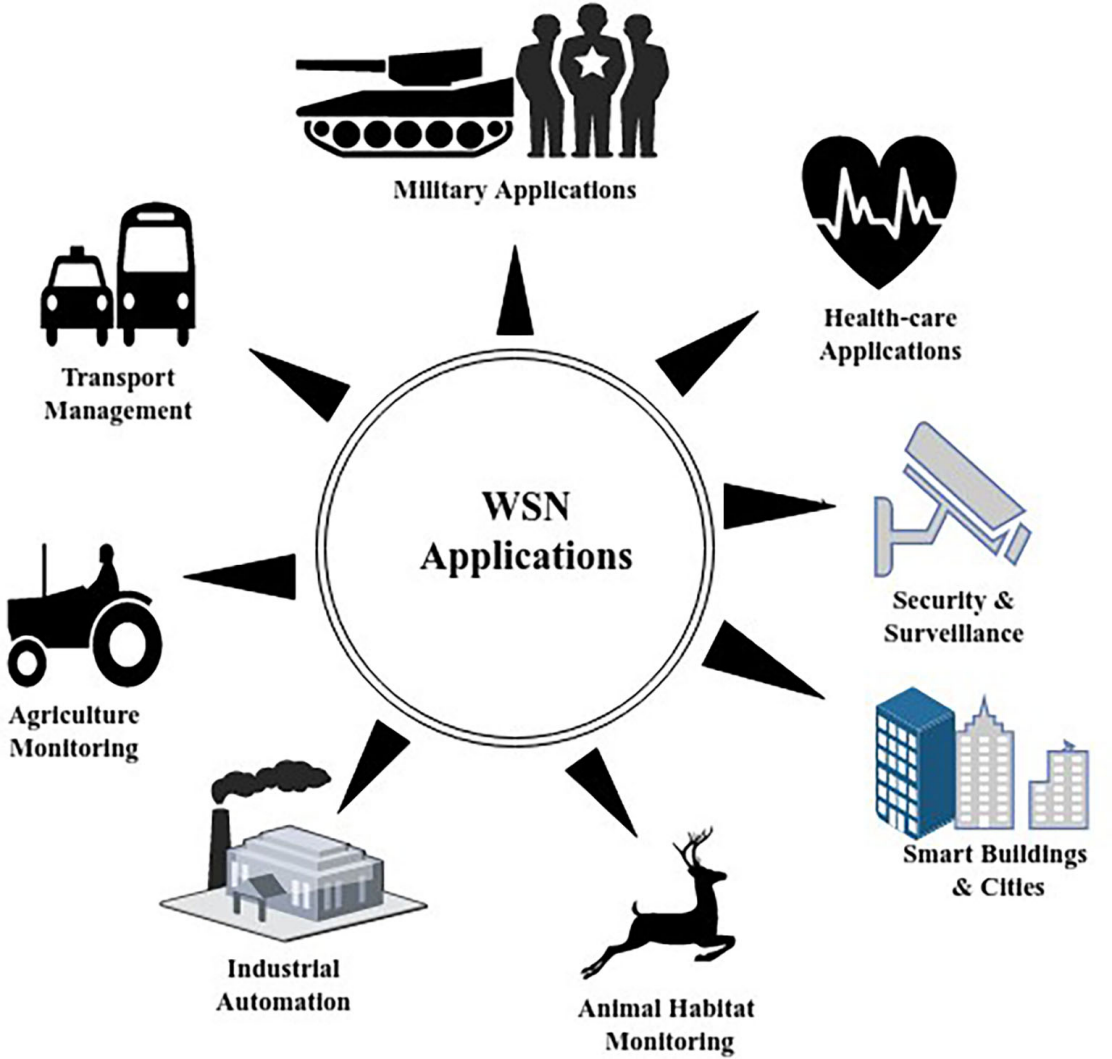
Applications of wireless sensor networks.

To attain a high level of efficiency in monitoring and control systems, importance is given to ensure the SNs are energy efficient (EE) and dependable data transfer in resource-constrained WSNs. A resource-constrained WSNs have nodes with less processing speed, limited data storage, limited bandwidth for communication, *etc* in comparison with wired sensor networks, where the sensor nodes are static which helps the user to upgrade the above-mentioned parameters as per the networks requirements. Extending network lifespan and boosting network dependability are two strategies to increase network reliability and decrease power usage of WSN nodes, respectively. ^5^ In WSNs, proper monitoring of a phenomenon depends on the collective data provided by the target cluster of sensors, not on any individual node. ^5^ Since WSNs are commonly used in many critical applications like territory tracking, ^6^ military applications, ^6^ health-monitoring systems, ^6^ and so on, the major constraint here will be energy conservation of SNs. Different applications of WSNs are shown in Figure 2.

Making the SNs EE will always remain the highest priority in today’s WSNs scenario. ^7^ Since the batteries of the SNs are not easily replaceable because of the deployment of the SNs in various terrains such as volcano detection in environmental applications, where the SNs are deployed which sometimes cannot be attended to. To maintain the energy of the sensors after their deployment in a sensor network, there are various energy-efficient protocols like “low energy adaptive clustering hierarchy (LEACH)”, “power efficient gathering in sensor information systems (PEGASIS)”, “threshold sensitive energy efficient sensor networks (TEEN)”, “adaptive threshold sensitive energy efficient sensor networks (APTEEN)” in WSNs. Such protocols would not only increase the sensor networks’ energy efficiency but also length of operation, throughput, and latency.

We have reviewed some enhanced EE protocols and a comparison is made with standard protocols in the article. These protocols have been categorized based on their application scenario. Even though the authors of the articles which have been reviewed here have made comparisons with standard protocols concerning lifetime enhancement, throughput, energy efficiency, packet delivery ratio (PDR), *etc.*, our main concern here is the energy efficiency of these reviewed articles. The primary aim of this review article is to emphasize the outcomes and the limitations of each of the protocols so that researchers can get into the detail of these aspects and come up with algorithms which are more EE and also design more reliable WSNs protocols.

### Definitions of some factors of energy efficiency

Table 1 below gives the definitions of the factors that determine how EE the protocols under consideration are listed in this paper.

**Table 1.** Definitions of the factors for energy efficiency in wireless sensor networks.

Factors	Definitions
“End-to-end (E2E) packet delay”	“The time taken for a packet to be generated at the sensor to the time it is received by the sink”
“Network Lifetime”	“The period before the first node in the network runs out of energy”
“Data reliability”	“The likelihood that an end to end communication will be successful”
“Sum of path length”	“The amount of energy used for multicast packet transmission”
“Multi-Level Facility”	“It is involved in cutting the total sum of path lengths to multicast members, allowing energy savings”
“Packet-loss recovery”	“The ability of nodes to recover dropped packets during network transmission”
“Energy Consumption”	“The overall amount of energy used by the network to carry out data aggregation, transmission, and reception”
“Packet Drop ratio”	“The ratio of the total number of dropped data packets by the total number of transmitted data packets”
“Average delivery ratio”	“The ratio of data packets received at the receiver end to that was initially sent by the sender”
“Transmission delay”	“The amount of time needed to push every bit of the packet into the wire is known as the transmission delay in a network”
“Average hops”	“The average hop counts as the distance that any packet must travel to reach the sink”
“Network throughput”	“Data transmitted from cluster heads to sinks and from nodes to respective CHs are both included in the total data rate that is measured across the network”
“Latency”	“The period between the moment the data is distributed and the time there are no nodes available to relay the data”
“Packet loss ratio”	“It is the proportion of total sent packets to packets that were lost”
“Number of Active Sensor/Operational Nodes”	“The total number of nodes that remain active or completely functional after network operations have been halted”
“Packet Delivery Ratio (PDR)”	“The proportion of packets that are successfully delivered to their intended location to all packets sent”
“Fractional Number of Void Nodes”	“The quantity of nodes that are outside the cluster zone and are unable to locate a CH for data packet forwarding”
“Optimal Number of 2-Hop Clusters”	“It is developed to reduce energy consumption for both intra-cluster and inter-cluster communications”
“Scalability”	“It is an important factor in designing an efficient routing protocol for wireless sensor networks”
“Sink Speed”	“Data transmission to the sink is measured as sink speed”
“Network Size”	“The total area of the network in which the sensor nodes are distributed”
“Delivery delay”	“The period between the packet's creation and delivery to its destination”
“Stability Period”	“The period between the beginning of network operation and the first sensor's death”
“Instability Period”	“The period from the first sensor's death to the last sensor's death”
“First Dead Node (FDN)”	“Defined as how many rounds pass before the first sensor is dead”
“Half Dead Node (HDN)”	“Defined as how many rounds pass before half the number of sensors is dead”
“Last Dead Node (LDN)”	“Defined as how many rounds pass before the last sensor is dead”
“Number of CHs per Round”	“The number of nodes that would transfer information compiled from their cluster members directly to the sink”
“Number of Alive Nodes per Round”	“The number of nodes whose energy has not yet been fully used up”
“Packet Delay”	“The time required by a packet to reach from source to destination. It is calculated by dividing the distance from source to destination by the speed of light”
“Hop Stretch”	“The ratio of the typical hop counts to the hop counts along the shortest path between the source and sink nodes is known as the hop stretch”
“Load balance of the network”	“By lowering energy usage, load balancing can be utilized to increase the lifespan of a sensor network”
“Residual energy”	“By adding up the energy used up while the sensor node was in each condition, it is possible to determine the remaining energy of the node”
“Network overhead”	“The total amount of bandwidth, energy, memory, time, and other resources that each sensor node in the network is using”
“Portion of Living Nodes”	“The ratio of nodes that may send messages to sink nodes is known as the part of living nodes”
“Energy balance”	“It costs some node energy to achieve the energy balance between the nodes”

## Literature review of energy efficient protocols for WSNs

In this section, previous review articles based on the energy-efficiency protocols for WSNs are discussed. The authors have thoroughly compared the EE techniques that are already in use. This section assists in understanding the various factors of the protocols which helps them in being EE by reviewing the already reviewed literatures. This review’s major objective is to inform readers about the current protocols that help WSNs.

Recent developments in sensor network routing mechanisms are discussed in Ref. 8. Appropriate categories, like data-centric, location-based and hierarchical, are used to discuss each routing protocol are compared from earlier literatures. The main goal is to clarify WSN routing methods and identify unresolved problems that may be the focus of future studies. This research is distinguished from earlier studies on sensor networks as it focuses on data-centric routing approaches. The protocols for hierarchical routing are described which is again based on data-centric routing. The location-based routing in sensor networks is illustrated in Ref. 8.

A strong emphasis on energy efficiency is considered in Ref. 9 and it provides a detailed discussion of topology control strategies so that battery-powered WSNs can extend their life comparing the already existing articles. Important topological control strategies are investigated in Ref. 9 to give light on how effectively energy efficiency is accomplished through these designs. The algorithms studied are further categorized based on the energy saving technique they implement and assessed by the disadvantages they provide so that designers can choose a better result that suits the applications. This article explains and examines the representative “atypical hierarchical routing protocols” and its benefits and disadvantages have been studied because the idea of “network lifespan” is frequently considered to study the algorithm’s overall outcome. Many research questions for attaining energy efficiency through protocol based on topology control on the findings are noted. The most recent advancements are discussed and update the data from earlier existing publications. Energy-saving clustering and mode scheduling techniques are included so that this work gives a more thorough survey. “Network lifetime”(NL) notion in the literature of protocols based on topology control exhibits notable variations. In contrast to past research, this work focuses on the factors that enable energy savings and increased network’s longevity for WSNs. Furthermore, unlike those that cover both centralized and distributed systems, the protocol based on topology control provided in this study are exclusively distributed approaches. ^9^

The “cooperative diversity-enabled medium access control” (MAC) protocols for LANs and WSNs are studied in Ref. 10. With the help of the transceiver pair, adjacent nodes function as”virtual multiple input multiple output” (VMIMO) systems in cooperative diversity protocol, sending different iterations of a packet to the receiver over different fading channels. The wireless link’s spatial diversity can be used in conjunction with several replicas of same packet to recover the original one, increasing reliability. By successfully coordinating transmissions between parent, associate, and destination nodes, MAC protocols make a substantial contribution to making this concept a reality.”Channel state information“(CSI), which is supplied to a routing layer for partner selection, can be collected across neighboring nodes using cooperative MAC protocols. While the majority of the initial research focused on physical layer approaches, researchers have now looked into supporting cooperative diversity in top layer protocols, specifically in the MAC layer. The first focus was on creating cooperative MAC protocols based on the IEEE (Institute of Electrical and Electronics Engineers) 802.11 standard for Wireless LANs (WLANs). The application of cooperative diversity in a variety of technologies, such as WSNs and cellular networks, has recently been the focus of research. In this paper, advances in developing cooperative MAC protocols both to WLANs and WSNs are described. ^10^ The outcome of this study show that the energy efficiency can be enhanced by having proper placement of relay between sink and source, distance among the sink source and in-between nodes can be made optimal. The drawbacks are that selection of optimal placement of a relay is a complex process and the trade-off between energy efficiency and reliability should be considered for the cost efficiency of the networks.

Article ^11^ provides a complete review of”atypical hierarchical routing“(AHR) for the first time. In-depth evaluations of several existing logical topologies are provided together with the peculiar WSN hierarchical routing. The benefits and drawbacks of several AHR protocols are examined regarding their noteworthy performances and application scenarios. The remaining issues using hierarchical WSN structure are also presented. The goal of this study is to offer helpful advice to system developers on how to assess and choose the best logical topologies and hierarchical routing techniques for certain uses. A categorization approach for WSNs’ unusual hierarchical routing is provided. This offers readers a fresh viewpoint to comprehend this style of routing. This division of atypical hierarchical routing into four categories based on logical topologies is novel. This is the first attempt to give an in-depth analysis of WSNs’ unusual hierarchical routing methods. The characteristics, benefits, and drawbacks of many traditional and contemporary atypical hierarchical routing algorithms are discussed in this study. A thorough evaluation of the general performances and use cases of various atypical hierarchical routing technique is described. A few unresolved problems in this area of study are listed. This research topic continues to advance as new research directions for researchers. ^11^

The study in Ref. 12 seeks to fill in the gaps and offers a current analysis of the sink mobility problem. Reviewing mobility management plans from an evolutionary standpoint by referring to already available literatures is its key contribution. “Unrestricted mobility” (UMM), “Uncontrollable mobility” (UMM), “Location-restricted mobility” (LRM), and “Path-restricted mobility” (PRM) are the four categories into which the related systems have been categorized. The proposed taxonomy is followed by an explanations of a number of typical solutions. To aid readers in comprehending the progression of growth within the category, the association between solutions is shown, along with extensive descriptions and in-depth analysis. By doing this, a few open issues that receive little attention or have not yet been thoroughly investigated are discovered, in addition to some prospective extensions based on recent research. It offers a comprehensive overview of mobility models that have also been often used in WSNs. It provides updates on current advancements in research and suggests that sink mobility management methods in WSNs should be viewed from an evolutionary perspective. By analyzing the advantages/defects of existing solutions and the relationship between them, it reveals potential extensions based on recent research and also several open areas which have gone unnoticed up to this point. The mobility concepts and features in WSNs are also described. The control of sink mobility is compared in this investigation. The conclusions and recommendations for additional study are outlined. ^12^

A detailed analysis of “Ant Colony Optimization” (ACO)-based routing protocols in WSNs” for the first time is considered in Ref. 13. Sorting different routing algorithms from earlier review articles into categories is the initial step. Second, one of most significant ACO-based routing methods are described, discussed, and qualitatively compared. Also highlighted are a number of outstanding WSN design issues. The primary goal of this survey is to critically review the most widely used ACO-based routing strategies developed for WSNs. This will assist in educating a huge audience about its existence. Various WSN ACO-based routing protocol types have also been listed. The ACO-based scheduling algorithms in WSNs are now categorized for the first time. It will be simpler for readers to comprehend these regulations as a result. Along with defining illustrative ACO-based routing protocols in WSNs, a summary of their benefits and drawbacks, and an evaluation of these protocols using a few metrics are also provided. This can be used by application developers to locate appropriate and additional solutions. Based on the most recent advancements in WSN technology, several research problems are highlighted and indicate some protocols based on ACO routing in future. This will advance the growth of this area of study. Finally, some unresolved problems and difficulties are also discussed. ^13^

The article ^14^ demonstrates the need for further focus on QoS-aware clustering. Furthermore, it is necessary to define how clustering can enhance user”quality of experience“(QoE). For intelligent systems to be able to support a variety of scenarios, it is crucial to understand the needs of the users. The implementation of clustering techniques for IoT systems in 5G networks is also discussed in this research. Numerous issues associated with using clustering techniques in a 5G setting for IoT are which are noted from the pre-existing articles are presented and talked about. One of the main strategies for green computing in WSNs is clustering, which may be used in a variety of systems. By just partially engaging the network’s sensors, such strategies can increase a WSN’s lifespan. Reviewing previous research from a QoS standpoint is necessary. Since the IoT concept was introduced, related techniques have been extensively used to assist people in their daily lives. IoT systems’ high diversity and usability set them apart from WSN systems. Examples of IoT systems include connected cars, smart homes buildings, *etc.* The trend toward wireless is the development of future networks (5G networks), and the migration of current IoT systems to such cutting-edge communication platforms. The distinctions between WSNs and IoT are studied in this paper, as well as the difficulties in integrating clustering algorithms into 5G-based IoT systems are investigated. ^14^

The current advancements in WSNs are examined in Ref. 15, covering their applications, design restraints, and lifetime prediction approaches. NL maximization strategies is introduced, followed by the presentation of a collection of definitions for the NL design target used for WSNs. A few design guidelines with examples are then provided to illustrate the potential improvements of the various design criteria. Considering the most recent developments, a concise taxonomy of smart WSN applications is offered. A thorough list of the design restrictions for WSNs is given keeping mind the flaws faced by the earlier review articles. The definitions of NL are presented in general. The most current NL maximizing techniques are evaluated critically, and their efficiency, objective functions, and constraints functions are examined. ^15^

In Ref. 16 the focus is on closely examining currently available communication standards and protocols to find any security flaws. The literature-available countermeasures to make sure the safety of protocols for communication against malignant activity have been studied, and they also present research problems and open research topics. The WSN routing protocol serves as the focal point of this paper’s study, but it also divides it into three categories according to the kinds of attacks each was created to withstand: There are three categories listed: “intrusion detection systems” (IDSs), “reactive solutions” (RSs) and “proactive solutions” (PSs). Finding the weak and strong countermeasure points, leads to the development of more secured protocols for WSNs. Developing a responsive system that could detect intruders, but also help the networks in intrusion recovery and avoid service disruptions is one of possible research routes in construction of safe WSN systems. An in-depth examination of network layer assaults launched against the protocol for “routing protocol for low-power and lossy networks” (RPL) routing concludes this study. The effect of specific assaults on a network’s performance is calculated by using Cooja network simulator. Lastly, new network layer security research possibilities are discussed, as well as how to use Cooja as a baseline to develop novel WSN system defenses. ^16^

Different EE routing protocols are taken into account in Ref. 17. This study aims to determines benefits and downsides of already reviewed articles. This study provides a suitable selection of an EE algorithm for various WSN applications by taking into consideration a number of variables. The decentralized hierarchical cluster-based routing algorithm makes use of both the routing based on cluster algorithm and the multi-criterion clustering algorithm. These algorithms run simultaneously. The “hierarchical energy efficient clustering” (HEEC), which improves high energy utilization and intends to prolong the life of sensor networks. In WSNs, the”clustering arrangement energy efficient routing protocol“(CAERP), that performs better than traditional clustering methods, intends to cut back on unnecessary energy use. The”centralized energy efficient distance“(CEED) routing protocol’s distributed CH selection mechanism is used to cluster and balance energy in SNs. The importance of this study is to discuss alternative routing algorithms while balancing energy usage and NL for WSNs. ^17^

Hierarchical EE routing algorithms based on swarm intelligence and traditional methods are discussed in Ref. 18. The routing protocols belonging to both groups can be classed based on aggregation of data, energy efficiency, QoS, location awareness, fault tolerance, query-based, multipath, and scalability. A thorough study has been conducted on the hierarchical EE routing techniques disclosed from 2012 to 2017. A fundamental explanation and practical foundation are provided along with a taxonomy of protocols for routing. The protocols are analyzed in detail, including details on their objectives, classifications, methods, benefits, and potential uses in the future. Hierarchical routing algorithms, such as energy consumption, categorization location awareness,”quality of the service“(QoS), data aggregation, scaling, fault tolerance, query-based protocols, and multipath are thoroughly studied in this review. This work outlines a few of the unresolved problems in this field of study. Finally, fresh research avenues have been suggested to advance this field. ^18^

A comprehensive analysis of existing protocols that is suggested for energy consumption, PDR, NL, and route creation time is presented in Ref. 19. These tactics are derived from a variety of different games. It qualitatively contrast the major characteristics, advantages, and disadvantages. Three parameters were considered while choosing the articles to include in the survey. Along with the publications, seminars, and publishing year where the papers were published, the implication of the three different types of game theory are provided. The three subcategories of game theory are cooperative, non-cooperative, and evolutionary game theories. It doesn’t necessarily follow that a routing system will become considerably more appropriate over a certain application just because it adopts a new gaming style. Future research directions are also highlighted, along with a few significant unresolved issues. ^19^

In addition to discussing the EE time synchronization over WSNs implementation problems, ^20^ also looks at the features of the transmission state within the deafness as well as packet collision. It is specifically a thorough audit that includes tools, drawbacks and areas of interest from earlier related work inside the transmission state. The study assists researchers in three areas: (1) preventing packet collision during communication in WSNs, (2) preventing deafness that occurs during transmission in WSNs, and (3) increasing data collection throughout transmission states in WSNs. Additionally, it suggests a few pertinent open problems as ideas for ongoing investigation. An unusual center is offered to contain tools, points of interest, and downsides on related works in the transmission condition. The work contributed to research by identifying the primary problems and obstacles to energy efficiency in WSNs, classifying energy efficiency schemes according to the needs of the applications, and making general conclusions regarding all energy efficiency schemes. ^20^

This is the initial offering of a complicated mathematical definition of EE in static WSNs. Attributes relating to the routing mechanism and pattern of traffic flow are displayed. ^21^ The underlying cause of the “Hot Spot Problem” will be identified. “Hot Spot Problem” can be defined as draining of energy of the nodes closer to the sink more drastically because these nodes will be involved in communication within the network, and they get isolated. Additionally, based on the examination of the energy consumption characteristics, the concepts related to EE, such as the EE Tier, EE Perspective, and the EE Means are offered and described. The planned EE of WSN techniques from 2002 to 2019 is tracked and thoroughly examined. The “mobile node assistance scheme” (MNAS),”energy efficient MAC” (EEMAC) protocol, the “energy efficient routing scheme” (EERS), the “energy efficient clustering scheme” (EECS), and the “cluster-based compressive sensing data collection” (CSS), respectively, are divided into five main categories. The representatives that were frequently highlighted in recent years are presented in tables along with an analysis of each category’s design principle. The relationships between the five categories, as well as those between each one and the EE Tier, EE Perspective, and the EE Means are all carefully examined. Also specified is the context in which each of them should be used. A thorough statistical analysis is also done for each category. Finally, the potential and constraints that WSNs currently face in the context of new computing paradigms are highlighted, along with several workable research areas for EE of WSNs. ^21^

The traditional and contemporary protocols are divided into categories based on the following factors in Ref. 22: (i) network structure; (ii) data exchange; (iii) usage of location information; and (iv) support for quality of service (QoS) or multiple pathways. This article’s goal is providing a comprehensive presentation of traditional protocols comes under each of above classifications, the traditional as well as modern ones, highlight their key attributes, providing a discussion about both specific and general issues raised, and pinpoint areas for further research. In comparison to earlier evaluations of a similar nature, the work described in this paper offers both an updated and a more thorough analysis of EE routing algorithms. Additionally, it both expands on the taxonomies that already exist and takes more performance factors into account for the analysis of the procedures under review. Comparisons based on performance measures and outcomes are also listed. The study’s findings are then examined, conclusions are taken, and unresolved research questions are listed. ^22^

An in-depth examination of “WSN hierarchical routing protocols” is the main emphasis in Ref. 23. The hierarchical protocols are classified based upon their routing techniques. This research compares several hierarchical routing methods. As a consequence of routing in WSNs and its significance in the study, an effort is made to offer a complete analysis of various routing techniques and their implications on the performance of WSNs. These protocols are grouped under tree-based, chain-based, area-based, or grid-based networks. The main goal here is to serve as a guide for comparing the most pertinent baseline hierarchical routing systems. A more in-depth examination of the leader selection criteria, hierarchical structure, and cluster construction process. The consequences of energy load and the limitations of different routing methods are then carefully considered. In order to aid in subsequent research, this study examines alternative hierarchical routing methods for WSNs. These reviews and contrasts various parameters used in cluster construction for various updated LEACH versions in order to provide the criteria to be taken into account when creating a cluster and routing. The most significant state-of-the-art methods are carefully chosen to distinguish and emphasize the performance, advantages, disadvantages, and challenges of each routing technique. Finally, a thorough analysis of the most recent advancements in adaptive clustering for low-energy hierarchical routing is given, following the comparison of the many versions discussed in this study. ^23^

An exhaustive survey of WSN clustering methods based on “hybrid energy efficient distributed” (HEED) is explored in Ref. 24. The advantages and disadvantages are listed and develop a novel understanding of the extended HEED-based WSNs protocols. Both the WSNs protocol rotated unequal HEED and energy-based rotated HEED are considered in this survey. For the next assessment and construction of protocol based on HEED, a fairway with the evaluation of every focused WSN algorithms is offered. The objectives of this survey were to highlight the benefits and drawbacks of each WSN protocol and its adaptability to various environments, provide extensive, well-explained variants protocols based on HEED new researchers in one place, and make it simple for network engineers to choose the best protocol for their needs. ^24^

Various energy-saving techniques explored by various research communities in WSNs which reduce the node energy usage and enhance the network’s lifetime are examined in Ref. 25. Duty cycle, EE routing, EE medium access control (MAC), and error control code (ECC) are just a few of the energy-saving protocols covered. The duty cycle strategy uses the sleep/wake method to cut down on the nodes’ active time and preserve energy. The MAC protocols along with routing employs relevant energy-saving methods. This study also briefly discusses certain energy-saving approaches that are researched vastly for different ad-hoc networks, such as the use of topology management, transmission power control and directional antennas. The literature offers methods for reducing energy use and boosting a WSN’s lifespan. The many energy-saving strategies put forth by various researchers to lengthen the lifespan of WSNs are discussed in this paper. Energy-saving strategies used by other ad-hoc networks in addition to WSN are also discussed. ^25^

In Ref. 26 WSNs EE routing strategies, its description and comparison of nine types of protocols, including network architecture, next-hop selection, network topology, protocol operation, delivery mode, initiator of communication, application type and path formation, according to a new proposed taxonomy has been explained. Each class is examined, talks about its representative routing protocols (benefits, drawbacks, mechanisms *etc.*), and then compare based on various factors for relevant classes. The main goal was to provide an overview of these methods using a fresh classification scheme that was previously discussed. A classification and comparison of current routing techniques under each category, highlighting their benefits and drawbacks are illustrated. ^26^

The EE methods for WSNs that collect energy and an “environmental monitoring applications” (EMAs) is examined in Ref. 27. As a result of the dynamic deployment and communication problems related to EMAs, it covers the concentrating on the physical layer, WSN protocol stack, MAC, as well as network layer. The study looks at the data, network, and physical connection levels of WSNs for EMAs to see how new security flaws influence them. In order to comprehend security problems in EMAs, this study evaluates WSN network protocols, EE protocols, as well as energy recovery protocols. Additional discussions are made regarding the different simulation settings for EMA protocol designs, specifications of the design, QoS specifications, and network topology specifications. This study’s conclusion describes the safety problems with WSN for EMAs, including the risks just at network and nodal level as well as ways to mitigate and prevent them. ^27^

A variety of literature reviews on energy-optimized routing techniques are investigated in Ref. 28. According to the investigations, “fault tolerance with optimal relay node employing modified particle swarm optimization” (FTOR-ModPSO) and “fuzzy optimal clustering” approach have been taken into account.”Intra mobile agents“(IN-MA) are the foundation of the “particle swarm optimization with genetic algorithm” (PSO-GA). The goals are to reduce energy usage, boost packet delivery rates, and determine where SINK should be placed in a WSN. To use PSO-GA method based on the intracluster mobile agent to reduce power consumption and optimize the pathways between SNs and the sink node. Fuzzy logic can increase network existence without causing collisions. For WSNs, energy-aware QoS protocols are studied and existing energy optimization strategies for LEACH-based routing were covered. ^28^

Strategies for controlling the routing techniques and topology in WSNs are combined to study collectively in Ref. 29. Different parameters related to EE is studied from the earlier reviewed articles. To improve WSN performance at both the topology and routing levels, taxonomy of topology management approaches and routing strategies has been suggested. The fundamental tenet of providing a detailed analysis of each categorized category is to demonstrate how well-established and applicable its evolutionary methods are. In this review article, each technique or protocol was arranged according to the year it was reviewed, with a detailed description of the proposed work’s value to society, the particular approach or methodology chosen, and any shortcomings that might be fixed in subsequent research. Main focus is on analysis of never-before-reviewed graph-based techniques, specifically designed interference models, topology control in WSNs and associated algorithms. Insights on cutting-edge routing protocols, including traditional hierarchical routing, “particle swarm optimization” (PSO)-based, and “ant colony optimization” (ACO)-based routings, have also been offered. Quick comparison of solutions that have been suggested has been made, along with a discussion of the research gaps and unresolved problems, as well as new research directions for this area’s future advancement. ^29^

## Comparative study of enhanced energy efficient protocols for WSNs

In this section, we have listed suitable articles and their explanation based on the EE protocols and their outcomes. The comparison is based on the various factors defined earlier for the efficiency of the WSNs. Researchers can benefit from this paper as it lists many different enhanced protocols giving them a better understanding of these protocols along with their outcomes as compared to the standard protocols.

A “3-Dimensional real-time geographic routing” (3DRTGP) was developed and analyzed. ^30^ This offers three main features. First, the 3DRTGP, which offers a soft real-time capability, is suggested for 3D-deployed WSNs. To enable real-time operation, the protocol employs an adaptable “packet forwarding region” (PFR) and selects “fast-forwarding nodes” within the PFR. By restricting the number of forwarding nodes going down the path of the destination, PFR seeks to lessen channel contention and congestion. The delivery of an effective heuristic approach for such VNP in 3D WSNs is the second. This strategy makes it possible for the proposed protocol to function dependably even when there are vacant regions present. Thirdly, tweaking methods for 3DRTGP are offered so that the protocol can satisfy application requirements for latency and miss ratio. For instance, changing network density can satisfy an application’s requirement for a low miss ratio. Because 3DRTGP does not rely on beacon signals to gather data about neighbors, it uses less energy than the protocols compared in this article. The 3DRTGP is compared with”above below location aided routing” (ABLAR) ^31^ and 3D Greedy protocol. For a WSN with 1000 nodes, 3DRTGP uses 31% lesser energy for every packet over ABLAR and 26% higher energy than for the 3D Greedy protocol.

In contrast to the other two techniques, 3D Greedy does have a high packet miss ratio, which might not be suitable for all real-time applications. Also, the proposed protocol is not applicable to mobile SNs within a network.

“General self-organized tree-based energy-balance routing protocol” (GSTEB) is analyzed. ^32^ A scenario where the network regularly gathers data from a landscape where each node continuously detects its surroundings and transmits the information back to BS is studied. There are typically two ways to define NL: a) the time span between the start of network activity as well as the initial node’s demise. b) The time frame covering the beginning of network activity and the death of the last node. The first definition is the one we use in this paper. In addition, it takes into account two extreme data fusion scenarios: case (1) it is possible to fully fuse the data between any two SNs. Regardless of the amount of data a node receives from its offspring, each node transmits the same amount of data; case (2) fusing the data is impossible. Each relay node transmits a message that is made up of the total of the data it has detected and received from its children. Another straightforward method for balancing network load is provided by GSTEB. In reality, it is challenging to uniformly spread the load over all nodes in such a situation. The proposed protocol is compared with LEACH, ^33^ HEED, ^34^ PEGASIS, ^35^ “power efficient data gathering and aggregation” (PEDAP), ^36^ “tree based energy efficient protocol for informtion systems” (TREEPSI), ^37^ and “tree based clustering” (TBC). ^38^

Even though GSTEB requires BS to calculate the topography, which lengthens the delay and increases energy waste, these energy waste and delay characteristics are manageable while comparing the energy usage and transmission delay for data.

An enhanced “fault-tolerant clustering routing algorithm established on a gaussian network for WSNs” (FCGW) is proposed in Ref. 39. Here the “geographic adaptive fidelity” (GAF) technique will be used to partition the random sensors into virtual grids (clusters). To achieve precise energy usage and extend NL, each cluster will choose single operational node once at time as a CH node. Each CH node can be described as a Gaussian integer and interconnected to one another to construct a Gaussian network because WSN management must indeed be refined as a Gaussian network. A fault tolerance technique for CH nodes is suggested in this paper. Based on the Gaussian network’s symmetric link, redundant routing paths can readily take the place of the primary routing path in the event of failure, thereby improving data dependability and optimizing energy consumption. The studied protocol is evaluated with FT-LEACH, HEED ^34^ and PSO-UFC protocols. ^40^

This protocol gives high reliability in terms of data. Also, it has shown that the energy efficiency is high compared to the protocols considered here. *i.e.*, it consumes 48% of energy whereas the other protocols consume 70% of its energy. It is difficult to join long-distance nodes in a wireless gaussian network because the packet latency would increase.

“Robust and energy efficient multicast routing” (RE2MR) algorithm is studied. ^41^ RE2MR is an enhanced multicast algorithm that addresses shortcomings of topology, geographic, and hierarchical multicast protocols while utilizing their benefits. It articulates the path search problem as the “capacitated concentrator location problem” (CCLP) but also determines the multicast topology which reduces path length’s sum of multicast base node to multicast members by substituting the existing concept of the “leader node” with the idea of the “facility node”. As a result, a system parameter may be used to restrain the number of subscribers that every facility node can manage. By using a “trajectory-based lightweight hole detection” (TLHD) method, RE2MR takes into account potential holes in realistic WSN installations. The knowledge of a hole found by TLHD and the combo of an application in iteration of CCLP enable RE2MR to adjust the multicast topology to be EE with respect to average path length. RE2MR enhances energy efficiency while utilizing wireless medium’s broadcast nature and precise packet header design. RE2MR is compared with “hierarchical geographic multicast routing” (HGMR), “robust and scalable geographic multicast protocol” (RSGM), ^42^ and “multicast routing with branch information nodes” (MRBIN). ^43^

Comparing RSGM and MRBIN, with RE2MR, the latter has an overall reduced total sum length, by up to 57%. One finding is that packet loss (PL) overall methods lower as node density (ND) rises. This can be explained by the fact that a node is more likely to find neighbors who are closer to its destination when the ND is greater. RE2MR does have an overall delay which is close to 8% and 50% lower than MRBIN and RSGM, respectively. The entire sum of path lengths will increase if the TLHD technique is not used because packets from a transmitter to a facility node from a facility node to multicast members pass across the face of the hole.

“Range-based opportunistic routing” (ROR) is an enhanced version of “energy efficient opportunistic routing” (EEOR). ^44^ A brand-new routing system named ROR is proposed, short for “Range-based Opportunistic Routing.” The opportunistic routing principle, which refers to the “option of nodes that have the least distance to the destination,” became a part of the EEOR protocol in new routing strategy ROR. ROR aims to decrease energy usage, extends lifespan of a SN, boost data transmission, and decrease the volume of control messages. Decreasing the number of forwarding lists from a different selection that only contains the nodes which are physically closest to the recipient and to have the maximum energy is the primary objective of ROR. The ROR protocol is compared with EEOR, ^45^ “extremely opportunistic routing” (EXOR) protocol ^46^ which are protocols for opportunistic routing.

The suggested protocol uses about 2500Mj of energy as opposed to the EEOR and ExOR protocols, which use about 3500Mj and 6500Mj of energy, respectively. When comparing performance in terms of dropped packets, the ROR protocol outperforms its competitors.

An “effective bypassing void routing protocol, which is based on a virtual routing circle” (BVR-VRC) ^47^ is studied. An obstruction is encircled by edge nodes to form the framework of BVR-VRC. The BVR-VRC has two optimization modes: void and greedy. Where the greedy processing mode uses a greedy algorithm strategy to choose the relay node. The void processing mode is turned on when a void is found. Void region partition, virtual coordinate map, and void detecting are the three stages that help compensate for the void processing mode. The edge node’s virtual coordinates are established following introduction of the void processing mode. Then, the greedy mode is reinstated, allowing a greedy algorithm to choose these edge nodes that have virtual coordinates as the relay nodes.”greedy perimeter stateless routing“(GPSR) ^48^ is compared with the proposed protocol and the following conclusion is made.

Delivery ratios become less favorable in BVR-VRC but also GPSR when routing void size grows. Delivery ratio in BVR-VRC is greater than those in GPSR at all void sizes. Even though the first packet data meeting routing void has a substantial delivery delay, average delay both in BVR-VRC and GPSR rise as routing void expands, with GPSR’s delay rising more quickly than BVR- VRC. However, the advantage is to minimize the average hops and ensure that subsequent packets always use a greedy approach to choose the delay nodes. As a result, the overall working time’s average delay is decreased. Well after virtual coordinate mapping of BVR-VRC, the entire routing process adopts greedy forwarding. As a result, BVR-VRC uses less energy on average than GPSR does.

**Figure 3.**
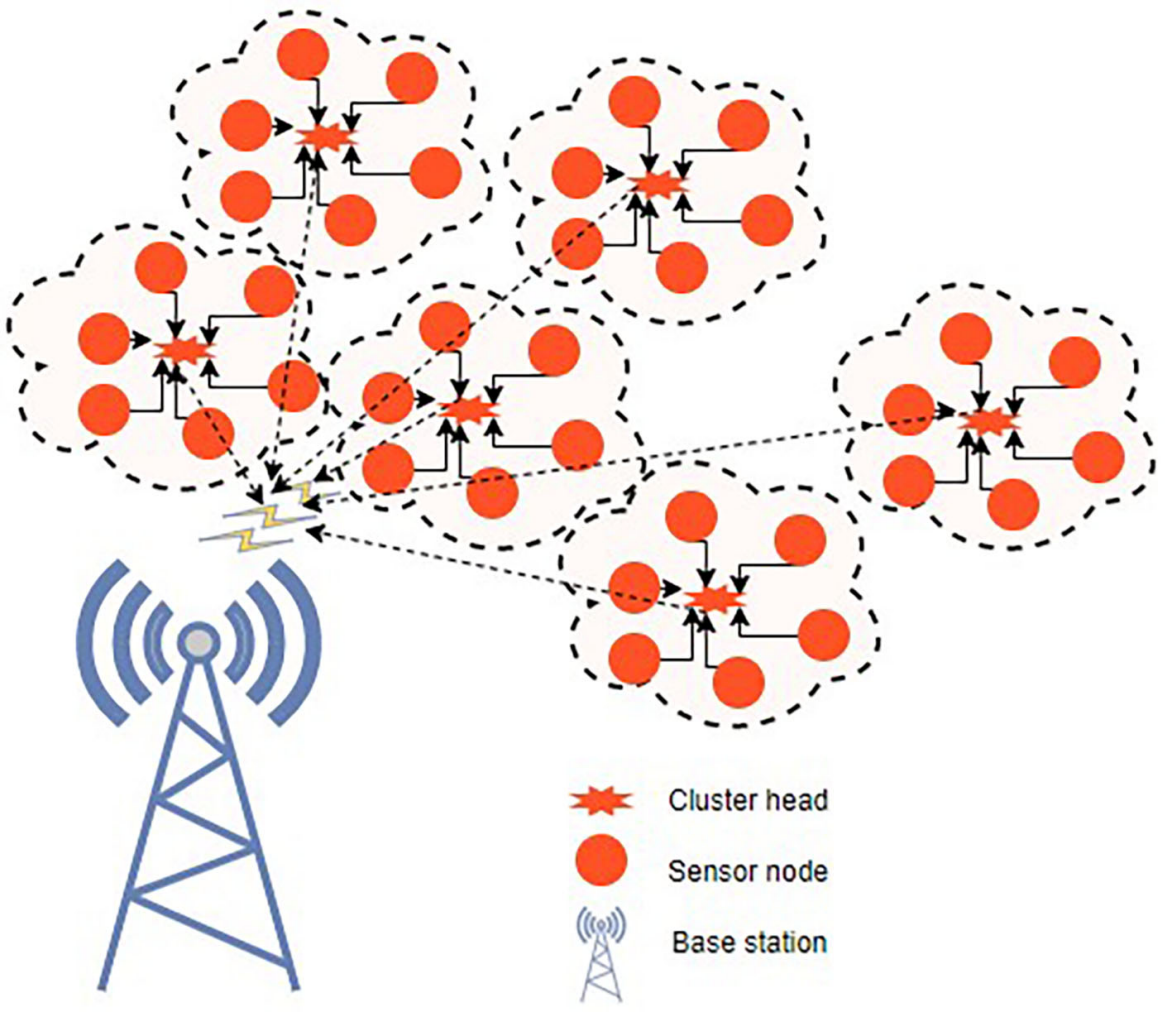
A clustered wireless sensor network. ^49^

“A more effective artificial bee colony (ABC) technique” is investigated. ^49^ Fuzzy C-means clustering and a novel, updated ABC technique are used to tackle the initial-round EE clustering problem. The clustered WSN architecture is shown in Figure 3. The best CHs may be chosen and clustering may be maximized if every node has the same energy level. To address the clustering issue of later rounds, an improved ABC algorithm-based optimal clustering method is proposed. The ABC algorithm’s honey source update principle is joined with the WSN’s characterization of CH energy, cluster position, as well as CH density in order to maximize clustering.

A routing approach based on an upgraded optimization of ant colonies is offered to find the best route out of each CH towards the BS. A busy/idle node based polling control scheme is created in the intracluster phase of communication to lower network energy usage and throughput of network will be increased. ABC-based clustered routing optimization is provided. According to results given by simulation process, the suggested method could effectively balance energy consumption, boost throughput, and extend lifespan of the network. The comparison of suggested techniques is made with “LEACH - centralized” (LEACH-C), ^33^ “fitness-value-based Improved grey wolf optimization” (FIGWO) ^50^ PSO ^51^ and “Artificial Bee Colony - SD” (ABC-SD). ^52^

In the metrics for total remaining energy, the suggested protocol performs well. This is due to the fact that by taking into account the energy component, both the recommended clustering and the ACO-based routing protocol discover the best result with respect to overall energy and energy balance. However, because the suggested method has only been investigated for stationary networks, it will need to be appropriately implemented for mobile WSNs.

A “dynamic cluster-based static routing protocol” (DCBSRP) is proposed. ^53^ With a superior E2E delay, lowest communication cost, maximum throughput, and lowest packet loss ratio, the recommended load balancing system is efficient and increases the lifespan of the installed WSNs. Additionally, the following measures have been adopted for the proposed scheme’s implementation: 1) Every SN is linked together. 2) A hybrid DCBSRP routing system is suggested and put into practice. 3) Static routing works for the duration of the specified time. 4) The studied method helps to sustain the life of the network with poor network communication and computation costs, and load balancing, very low packet loss ratio, rapid throughput and very low E2E delay. The “dynamic duty cycle (DDC) scheme”, ^54^ “EE connected coverage scheme”, ^55^ “optimal clustering in circular networks scheme” (OCCN) ^56^ and “distributed optimal on-line task allocation algorithm” ^57^ are compared with DCBSRP.

The suggested routing protocol minimizes network overhead and prolongs the network lifespan. It shows how long a single node may endure in the network when the suggested algorithm is in use. Furthermore, the observed regular node participation percentage was 95.9%. The effectiveness of the studied strategy in increasing the lifetime of network was astoundingly huge since the creation of micro clusters not only manages the network’s load but also appears to serve a promising function in enhancing the lifetime. Due to the clusters’ unicast communication, which reduces the possibility of network overhead, the latency was discovered to be relatively constant. The initial deployment of the DCBSRP routing system is difficult, however this is a one-time operation with long-term benefits.

“An efficient 3D WSN routing scheme” (3D cluster protocol) is suggested. ^58^ An innovative routing approach that combines SN scheduling with clustering routing in order to improve energy efficiency and lengthen NL n the 3D WSN is introduced. The set with fewest number of SNs using an”improved genetic algorithm“(I-GA) can be located, which makes use of unnecessary SNs of the 3D network to discover and switch them off. In order to find the optimum SNs to swap the energy-drained nodes, a node wake-up technique is also included. Due to the less-than-ideal wireless channel, the additional energy usage of the retransmission is also accounted for by this routing. This protocol is analyzed with main-3D ^59^ Leach-3D. ^60^

Fewer nodes are functioning at a time because of 3D-cluster protocol transfers data depending on the ideal node set, which significantly decreases energy usage. The authors modify the likelihood that SNs will turn into CH in the article, redesign intra-cluster and inter-cluster communication, and enable SNs surrounding BS to connect with BS directly without joining the cluster group. The network throughput decreases as the SNR threshold rises. This is because as the SNR threshold rises, channel quality requirements rise as well, increasing the likelihood that a link with relatively low quality may be disrupted. The frequency of data retransmission also will rise as a result, which consumes network energy and reduces NL. The network’s throughput will likewise be lowered proportionately at the same time.

A new “distributed 2-hop cluster routing protocol” (D2CRP) is suggested. ^61^ In order to balance energy usage on packet transmission and CH competition by minimizing the transmission distance, a distributed 2-hop clustering technique is developed. Employing 1-hop neighbor nodes as relays for transmitting data for each 2-hop cluster, a distributed intra-cluster routing approach is employed to achieve energy-efficient transmission in a cluster. To reduce overall transmission distance of each CH, a distributed inter-cluster routing technique is used to connect the CH nodes and send data to the BS in a chain-based manner. As a result, each CH can communicate with the BS using less energy. To decrease the amount of energy used for network transmission, the ideal cluster size of 2-hop clusters is proposed and discovered. This protocol is contrasted with LEACH, ^62^ PEGASIS, ^35^ R-LEACH, ^63^ and TTDFP. ^64^

After RLEACH, PEGASIS, TTDFP, as well as the planned D2CRP, the LEACH protocol was the one to fully use the energy. Following the same cycles, the LEACH procedure uses the greatest energy and has the quickest curve growth. The D2CRP has a rate that is approximately 52% worse than LEACH, 27% worse than R-LEACH, 18% worse than PEGASIS, and 2% slower than TTDFP. The suggested D2CRP may be tested in mobile node settings. For practical applications, it is also recommended to investigate developing the routing scheme established on widely adopted standard like ZigBee and Bluetooth.

“A delay-aware green routing protocol for virtual infrastructure” (DGRP) is analyzed. ^65^ To lower the delay in transmission of data, a method for building the virtualized hierarchy of rings is proposed that enables each SN to communicate data toward a virtual sensor (VS) node in a 1-hop communication. A ring maintenance system is also suggested as an answer to the hotspot issue. Without initially acquiring the position of the mobile sink *via* VS nodes, SNs can transfer data to the sink using DGRP. Instead, Routing based on angle, which aids the VS node in choosing the path with the shortest length, is used to transport the information to the mobile sink through the virtual ring. Therefore, it is suitable for applications that require quick responses. Different experiments are carried out to assess and compare the suggested protocol with existing protocols of critical performance characteristics, such as energy consumption and latency. The ring routing protocol (RRP), ^66^ and “grid-cycle routing protocol” (GCRP) ^67^ are compared with the proposed protocol and their results are briefed as follows.

Energy utilization of RRP is higher than DGRP because of communication cost required to collect the sink position data prior to data transfers. However, using DGRP, source node sends information to the VS node, which then propagates farther toward the sink using routing based on angle within the ring. Because of its single-ring shape, RRP has the highest energy usage. This problem is solved by DGRP by building several rings. It thus relieves pressure upon SNs that frequently need to gather the sink location data for data transfers. Even though GCRP adheres to a set of guidelines to modify the sink position information, due to the mobility pattern of the sink, it uses a little bit more energy than DGRP. Dense sensor networks can use the suggested routing protocol. Future work will involve adapting DGRP to accommodate numerous mobile nodes to significantly enhance performance of data delivery.

“Destination-oriented routing algorithm” (DORA) is a novel multichain routing technique that is put forth. ^68^ In order to determine the appropriate cluster size of WSNs, DORA is built by taking into consideration both the actual communication distance and the packet forwarding direction in each node. This concept uses mathematical analysis to construct a cluster division that increases communication range and establishes the exact transmitting distance between any two nodes. DORA lowers the transmission energy needed for every routing channel by preventing the formation of the extended chains that might happen when using PEGASIS. It is compared with PEGASIS protocol ^35^ and “random projection-polar coordinate-chain routing” (RPC). ^69^

The average lifetime performance of DORA is over two times better than that of PEGASIS since PEGASIS’s energy consumption is greatly offset by its unexpected detours and unnecessarily lengthy chain routing pathways. With the polar routing architecture, RPC shortens the transmission path and outperforms PEGASIS in terms of lifespan performance. For different network sizes, DORA outperforms RPC in terms of lifetime by 60%, demonstrating that it can more efficiently shorten the routing path to the sink with a superior transmitting radius and polar angle.

**Figure 4.**
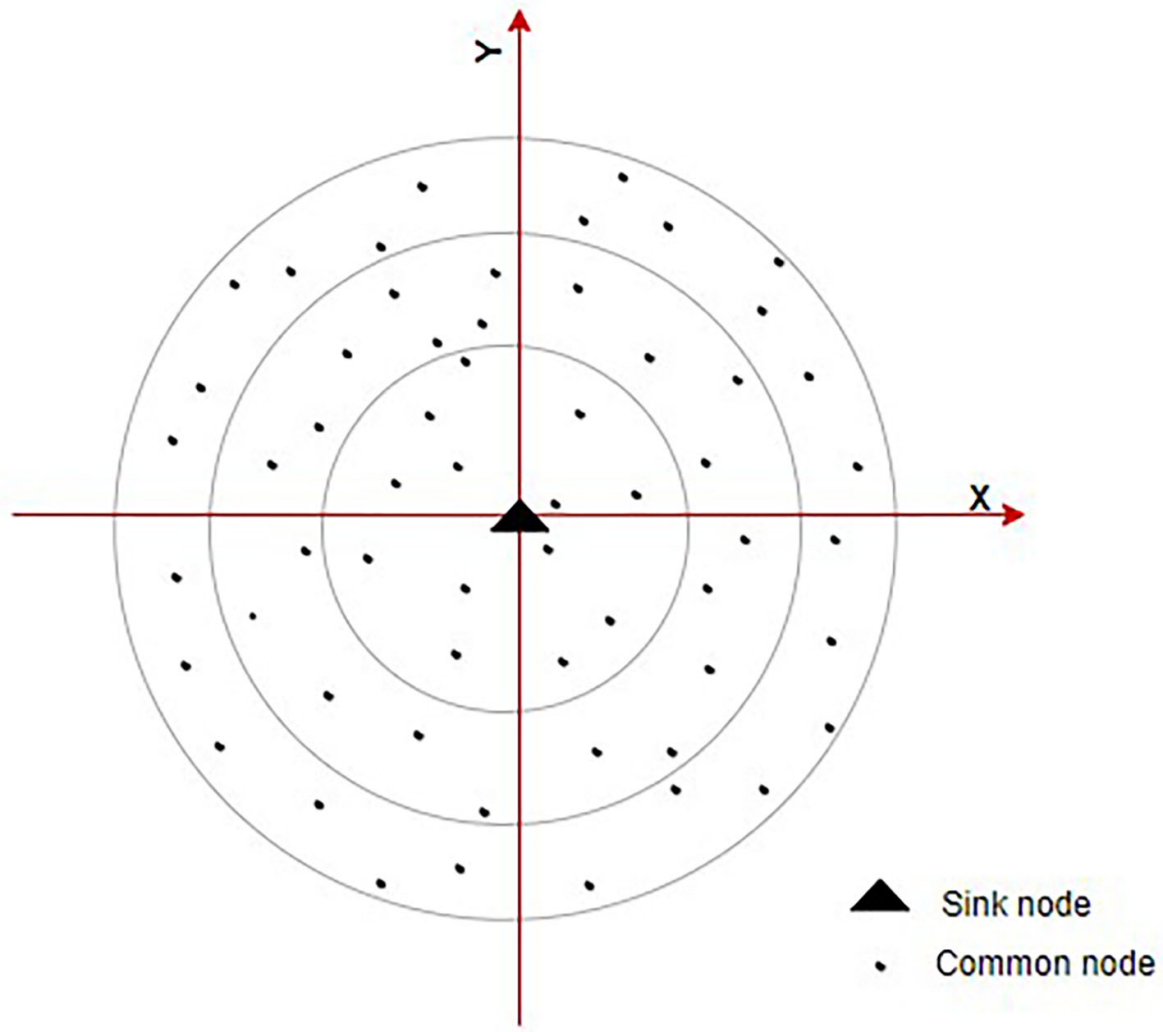
Ring communication topology. ^70^

A routing protocol for “energy efficient heterogeneous ring clustering” (E2HRC) is proposed. ^70^ This paradigm, nodes are grouped into various layers according to their relative placements. Additionally, different ring domains are split according to various levels. Figure 4 shows the ring communication topology. This lowers energy required for receiving and sending data by requiring the next node in one hop with the best direction angle to be chosen during interring domain communication. An energy balance-based routing protocol is provided, along with a clustering algorithm for it. The network is divided into various-sized heterogeneous clusters as according to node remaining energy and relative node location inside the cluster using a cluster probability model. Heterogeneous clustering and CH rotating processes are being used to balance node’s energy utilization and prevent the development of a network energy hole. Detailed explanations of the EE heterogeneous ring clustering-based E2HRC routing strategy are given, along with route creation, route maintenance, messages for clustering and clustering rotations.

The suggested E2HRC and RPL are contrasted. ^66^ When the original RPL was utilized instead of the routing algorithm suggested in this paper, the fluctuation range of node energy usage curve with in WSN was higher. A few energy balanced E2HRC routing protocol phases, including CH ratio calculation, ring domain communication establishment, and probability factor determination, were examined. To maintain uniform in the CH distribution in the rings, CH selection and the probability factor adjustments method were implemented. The lifespan and energy usage of WSNs were properly balanced.

A new routing protocol is proposed, called “energy efficient and reliable routing protocol for mobile WSN” (E2R2). ^71^ A proposed protocol is hierarchical. Our top priorities are accessibility to the nodes and energy efficiency. Node mobility is considered during the routing decision-making process. This kind of routing seeks to maintain data packet transit over appropriate channels despite the mobility of nodes and eventual link failures. Mobility of the BS and the SNs in the routing considerations are considered. The network’s lifespan is extended by using the CH panel concept. The idea of BS feedback on data delivery in it is taken into consideration. This method makes sure that data is sent reliably to the BS; this is done by using various routes and switching between them as the BS sees fit. A modified probabilistic model is considered that can be utilized to choose the most effective path for data transfer. M-LEACH ^72^ is compared with the proposed protocol.

The nodes move more quickly because of the high mobility environment. This protocol performs better than M-LEACH. However, as more nodes are placed in the field, the energy usage grows as well. The increase in energy usage is brought on by the evidence that as the node number rises, so does the quantity of packet exchanges, which results in a higher energy cost. Once more, average communication energy rises in tandem with network area expansion. This is as a result of the higher energy cost of long-distance communication. Even with the suggested protocol, throughput drops off dramatically while data rate rises.

It is suggested to use “signal to noise ratio-based dynamic clustering for WSNs” as an effective and safe routing algorithm. ^73^ A hybrid “efficient and safe routing protocol developed using SNR-based dynamic clustering mechanisms” (ESRPSDC) is developed, which is a combination of SNR-based robustic clustering and routing pattern-based safety mechanisms. ^73^ Threshold energy and node energy are the main criteria in this article. The node is chosen as the CH if its energy exceeds the threshold energy. The cluster list will then be modified. A quick comparison of ESRPSDC with two well-liked routing protocols: LEACH and PEGASIS is made.

The typical energy use, measured as mWh. Whenever the network size is changed with 30% of the nodes being malevolent, all three methods cut power consumption, demonstrating the strength of their clustering. However, ESRPSDC showed roughly 50% higher reductions when compared to LEACH and PEGASIS. The proposed algorithm has to be optimized for both heterogeneous WSNs and energy consumption.

The proposed protocol is a “biologically inspired secure autonomous routing (BIOSARP)” system. ^74^ The layout of the suggested method is based on enhanced ACO. TelosB radio sensing board that are wireless SNs, also employ BIOSARP. TelosB is a low-power transceiver based on the CC2420 ChipCon chip. Ten TelosB nodes being deployed in the field to construct an extensive WSN testbed. TelosB’s lighting, built-in temperature, and humidity sensors are activated while conducting the experiment. The proposed protocol is compared with “Energy and delay model based on ant algorithms” (E&D ANTS) ^75^ and “improved energy efficient ant-based routing (IEEABR)”. ^76^

When compared to E&D ANTS, BIOSARP acquires a lot less routing burden. This is because BIOSARP avoids using forward and backward agents and analyzes the data packets as needed. Additionally, BIOSARP outperforms SRTLD because it doesn’t run the broadcast operation on each hop. Comparing the results of BIOSARP and IEEABR reveals that BIOSARP uses less energy and also that the enhanced ACO algorithm used by BIOSARP is the sole cause of the better results.

The “efficient dual-path geographical routing” (EDGR) protocol is suggested. ^77^ EDGR utilizes two node-disjoint anchored lists that travel via both sides of routing holes to achieve dual-path routing, which prevents data being transferred over the boundaries of routing holes. By sending every data packet to the destination across two paths, if possible, in greedy form rather than bypass mode, this shortens the routing time and balances the load. In the event of a node malfunction in relay area, EDGR offers an innovative alternative technique to choose an effective forwarder by including a random shift to the position of the sub-destination. Without additional communication overhead, such a strategy is practicable, reasonable, and EE. EDGR is expanded into 3D sensor networks and should provide energy-aware forwarding for routing hole detour. The performance of EDGR and its extension in a variety of communication contexts, including various communication durations, network densities, and routing hole widths is evaluated. “Energy-efficient beaconless geographic routing” (EBGR), ^78^ “Energy-efficient Multicast Geographical Routing” (EMGR), and “Power Adjusted Greedy- Cordinate Face(1)” (PAG-CFace(1)-PAG) ^79^ are compared with EDGR.

The energy utilization increases in all the protocols compared in this article. This is due to the routing hole which lengthens the data transmission path. The energy is less utilized by both EDGR and EMGR which is as per the EBGR standard. Despite this, the NL of EDGR and EMGR are increased which is more than EBGR by 12.8% and 6.5% respectively. Also the EDGR uses less energy in comparison with PAG-CFace(1)-PAG which is 10.5% below that of PAG-CFace(1)-PAG even though the communication time increases. For less communication duration also the suggested algorithm uses least energy.

A suggested protocol for “enhanced balanced energy-efficient network-integrated super-heterogeneous” (E-BEENISH) routing is studied. ^80^ A mechanism that took the distance between both the source and the destination nodes into consideration is created to circumvent the overly straightforward threshold setting of the suggested protocol. The problem of the node farther from the BS dying early due to its energy consumption is exacerbated worse when the node remote from the sink node serves as the CH. The suggested protocol chooses CHs when taking into consideration the distance factor, the average energy of the network, and the leftover energy to avoid the ping-pong effect. A normalized weighting constant is suggested to increase the NL and evenly distribute the energy and proportion of distance in the thresholds design more evenly. “Stable Election Protocol” (SEP), ^81^ “Distributed Energy Efficient Clustering” (DEEC), ^82^ and “Centralized Energy Efficient Clustering” (CEEC) ^83^ are compared with this protocol.

It is clear that the SEP protocol performs well in the beginning, but as the number of rounds increases, performance rapidly declines. The network’s lifetime has increased by roughly 60%, 40%, 15%, and 25% compared to protocols for comparison, respectively. E-BEENISH also has more total leftover energy than other procedures. Although the suggested technique extends the lifespan of heterogeneous networks, it also makes the procedure more difficult. The technique, however, does not account for the network jitter and delay brought on by the latency in the data transmission phase.

“Multi-threshold segmentation-based energy efficient routing” protocol (EERPMS) is suggested in Ref. 84. A connection among node clustering and multi-threshold image analysis transforms the difficulty of node clustering into the difficulty of choosing the right segmentation threshold. Combining the Otsu algorithm with node angle and number results in a cluster creation methodology based on the variation of node angle and numbers between clusters. Various communication models among nodes in various scenarios are addressed to prevent substantial energy consumption among nodes brought on by the multi - path fading model. To decrease node energy consumption and lengthen NL, a multi-threshold segmentation-based energy-efficient routing protocol is presented. It is merged with the multi-threshold segmentation-based node clustering technique and the CH selection method based on the optimal CH position. This protocol is compared with ^63^ Residual energy based - LEACH" (R-LEACH), ^63^ FIGWO, ^50^ and “Clustering Routing Protocol based on Fuzzy C Means” (CRPFCM).

The energy usage of our suggested EERPMS has consistently been maintained to a minimum. The number of loads carried by CHs in each cycle is essentially the same because the load balancing factor is considered during clustering. The highest residual energy is in the EERPMS network. The EERPMS protocol can also save up to 64.5%, 58.60%, and 56.15% of network energy when compared to the protocols taken for comparison.

The “energy-saving clustering by voronoi adaptive dividing (ESCVAD)” technique is suggested in Ref. 85. It is successful in realizing dynamic clustering of WSN. Additionally, it fixes two problems with the standard WSN routing protocol, namely the uneven distribution of cluster and SN node deaths. Based on thorough weighing of distance and energy, an optimization approach for CH selection is developed. By efficiently balancing the energy use between both the CH node and the cluster member (CM) node, this approach increases the NL In addition to accounting for the effect of the CH’s positioning on the transmitting energy consumption, it also takes into account the energy level of the cluster-own head. This proposed an approach for reliable working time optimization. From the CH election stage through the stable operation stage, the relation between control signaling and energy consumption via signaling are both optimized. Energy consumption may be made to be more stable and gradual by designing the operating stage appropriately. “Minimum Transmission Energy” (MTE ^86^), LEACH, ^87^ “Centralized-LEACH” (LEACH-C), “Stable Election Protocol” (SEP), ^88^ and TEEN ^89^ are compared with the protocol suggested.

ESCVAD uses a lot less energy during the first two phases of signaling contact than other protocols. The MTE Protocol does have the fastest energy consumption, and it is almost twice as much as ESCVAD. The residual energy of the ESCVAD protocol has the slowest declining trend and the least energy consumption rate. While signaling only uses 0.31 J, ESCVAD’s energy consumption for data transfer work is 199.69 J. First two stages of the other five regimens’ energy requirements range from 29.28 to 52.04J. This outcome may indicate that the ESCVAD significantly increases energy efficiency compared to the other five treatments. Sink node element can also be taken into account as a movable factor in the detailed analysis of ESCVAD in the future. WSN routing algorithm in a mobile environment with the aid of this architecture should be explained.

“Multi-hop routing protocol is built on game theory and coverage optimization (MRP-GTCO)” is studied in Ref. 90. To prohibit the data of cluster members from not being transferred owing to the selfishness of nodes will not become the CH in the current network, a punishment mechanism depending on node residual energy and node degree is developed. In a clustering game with a punishment system, the likelihood of nodes becoming CHs is examined. An original CH selection strategy based upon that CH coverage rate and remaining energy is given in order to achieve the consistency of CHs and lower the transmission energy usage across nodes. The best multi-hop relay select theorem for clusters has the objective of transferring intra-cluster data to BSs whilst CHs consume the smallest amount of energy under a variety of circumstances. “Localized game theoretical clustering algorithm” (LGCA), ^91^ R-LEACH, ^63^ and “energy efficient clustering algorithm based on game theory” (ECAGT) ^92^ are used for comparison of the proposed protocol.

The proposed MRP-GTCO protocol maintains a range of CHs between 6 and 10, and in most cases, this range can fluctuate around the ideal range of cluster - head (10 nodes), which will help to ensure that the proposed protocol can operate with minimal network energy usage and guarantee energy efficiency. The MRP-GTCO CHs are evenly distributed over the detection region, and the distances between them and other CHs or cluster members are reasonable. There are nine CHs, which is nearly the ideal number. However, MRP-GTCO can always send more data packets to the BS while the network uses the same amount of energy. As a result, MRP-remarkable GTCO’s energy efficiency is evident. There is still room for improvement in MRP-final GTCO’s node death rounds.

“A hybrid method called energy and traffic aware sleep-awake (ETASA)” is proposed by Ref. 93. In heterogeneous WSN, a hybrid technique for load balancing and energy efficiency has been presented.

**Figure 5.**
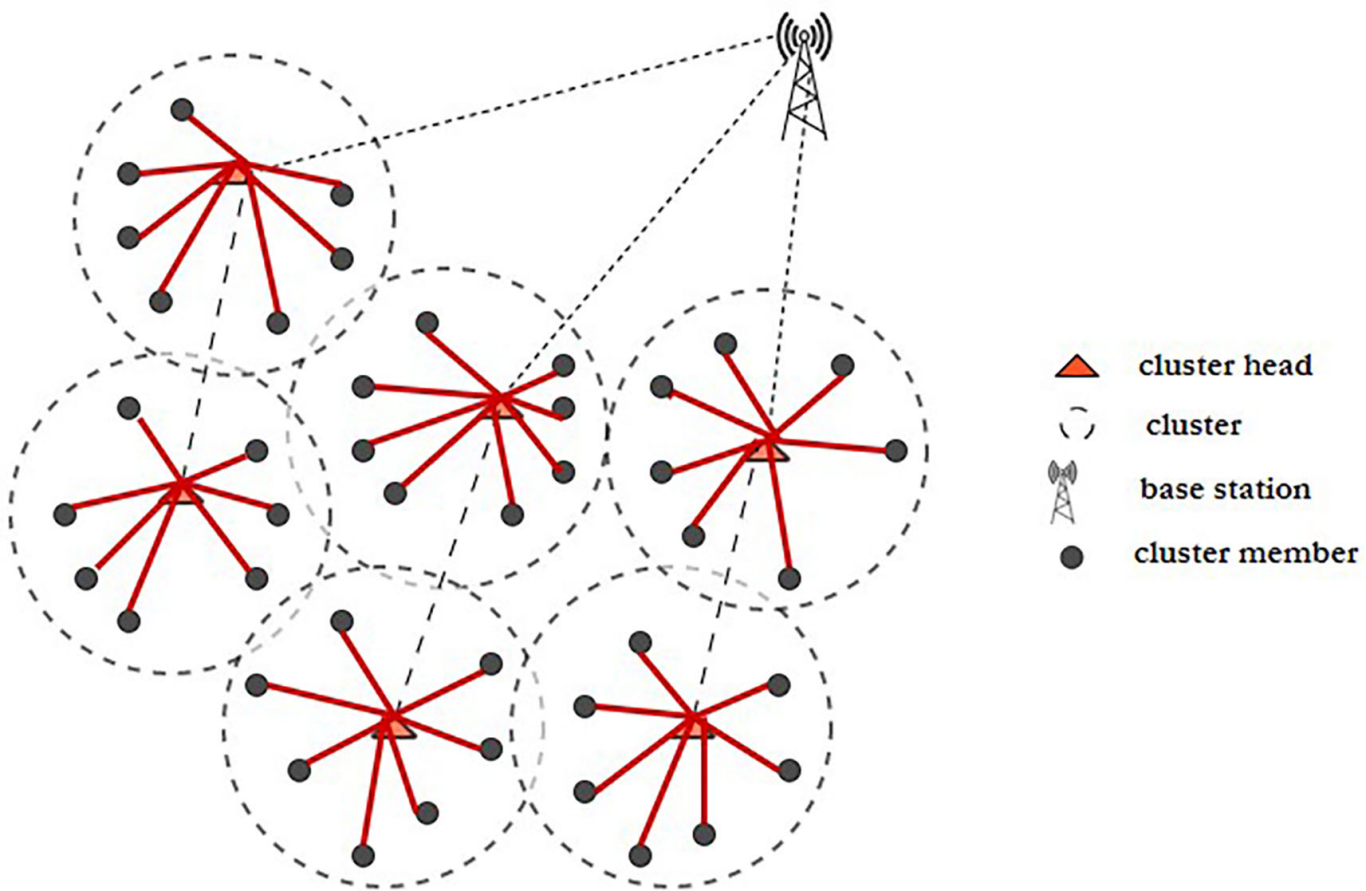
Clustering process. ^93^

Figure 5 demonstrates the clustering process. The possibility that a node will be chosen as CH is increased by the CH selection process, which favors highest enhancement of energy of node, the least amount of traffic, and also the most pairings. The load balance is enhanced by this CH selection technique. To decrease the number of permitted slots, idle monitoring in the network, and energy usage, the conventional “time division multiple access” (TDMA) scheduling by giving one slot to a group of pairs is broadened. Evaluation of the proposed method’s performance utilizing current cutting-edge baseline routing protocols. For that it is compared with the “Traffic and energy aware routing (TEAR)” protocol is presented in Ref. 94, and the “Sleep-awake energy efficient distributed“(SEED) algorithm. ^95^

Comparing the ETASA algorithm to TEAR and SEED, it has more energy left over. The CH selection approach employed in ETASA improves load balancing since it avoids choosing isolated nodes for CH roles, in contrast to TEAR, where the CH selection procedure primarily focuses on choosing high energy and low traffic nodes. The outcome demonstrates that the suggested ETASA has lifespan improvement against TEAR and SEED of 16% and 15%, respectively. The same network takes into account the traffic variability within the context of various zones.

“Mobile sink-based adaptive immune energy efficient clustering protocol (MSIEEP)” is studied. ^96^ MSIEEP was created to increase WSN longevity and lessen the energy hole issue. The positions of the mobile sink, the optimum number of CHs, and their placements are determined by MSIEEP using the Adaptive Immune Algorithm (AIA), which aims to minimize the total energy deficit through effective communication and overhead control packets forwarded by all nodes in the network. LEACH, ^33^ “Mobile sink improved energy efficient PEGASIS-based” routing protocol (MIEEPB) ^97^ are compared with the protocol in discussion.

It is clear that mobile sink improves the network’s lifetime and stability. Additionally, it boosts the network’s throughput while lowering energy loss. Additionally, comparing to MIEEPB protocol and rendezvous protocol, the suggested three moving patterns increase lifespan of the network by 56.79, 62.43, and 103.35% and by 30.35, 35.04, and 69.06%, respectively. Regarding the static sink, it can be shown that the proposed technique extends the stability time by 1315, 888.5, and 878.65 rounds, respectively, when compared with other protocols. In contrast, the stability period in the mobile sink case increased by 1875.2, 1732.2, and 1722.2 rounds, respectively, in comparison to the LEACH.

“Mode-switched grid-based routing (MSGR) protocol for WSN” is proposed. ^94^ A grid head is selected from each virtual grid that covers the entire sensing region. The MSGR protocol is then used to send data packets to the mobile sink. In its concluding portion, it suggests a method for limiting sink mobility and grid head re-election. An “energy-aware grid-based routing technique” (EAGER) for WSN ^95^ is compared with MSGR.

MSGR has more active nodes than the current protocol EAGER throughout the time period specified. This is due to MSGR’s ability to conserve more energy in nodes by alternatively putting data-disseminating nodes in the active or sleeping state. Additionally, additional node energy is lost as a result of the overhead rerouting in EAGER.

Due to the alternate switching of grid-head states, the proposed MSGR uses significantly less energy per node on average, avoids flooding of control packets for establishing routing paths (unlike EAGER), and doesn’t constantly reroute to find the shortest path. According to the methodology used, EAGER utilizes more energy per node than the way that was suggested. Future work on this project can be expanded by employing several mobile sinks.

“An optimized protocol called straight-line routing” (SLR) is proposed. ^98^ A unique random-walk routing system that we suggest is straight-line routing (SLR). This is really effective and easy to implement. Together, it considers low energy consumption and a high probability of successful path discovery. Monte Carlo simulations support the effectiveness of the strategy in this regard. Meandering pathways used by RR waste energy, and the packet payload contains a log of the nodes it has visited. RR does not choose visited nodes as a result, but it does not result in better searching directions. SLR, like RR, performs best when there are so few occurrences, as was already mentioned. In a WSN environment, this makes sense because nodes typically only form a small number of information requests (emergency events) when watching a region. By accounting for multiple beginning orientations, it also raises the baseline SLR. Then, it notes that reversing the initial direction might be a smart move if it goes away from the desired location. SLR expects a random-walk-style protocol to be more scalable and energy-efficient as a result. It is compared with “rumor routing” (RR). ^99^

The overall energy cost with a transmitting range of 50 can be decreased for RR because of hop count for all SLR techniques is lower than those for RR. Notably, all ratios were below 50% for small networks (transmission range of 50). Due to the possibility of numerous unreachable cases, this performance is not regarded as satisfactory.

We discuss the Q-DAEER algorithm, “an innovative Q-learning-based data aggregation-aware EE routing protocol”. ^100^

**Figure 6.**
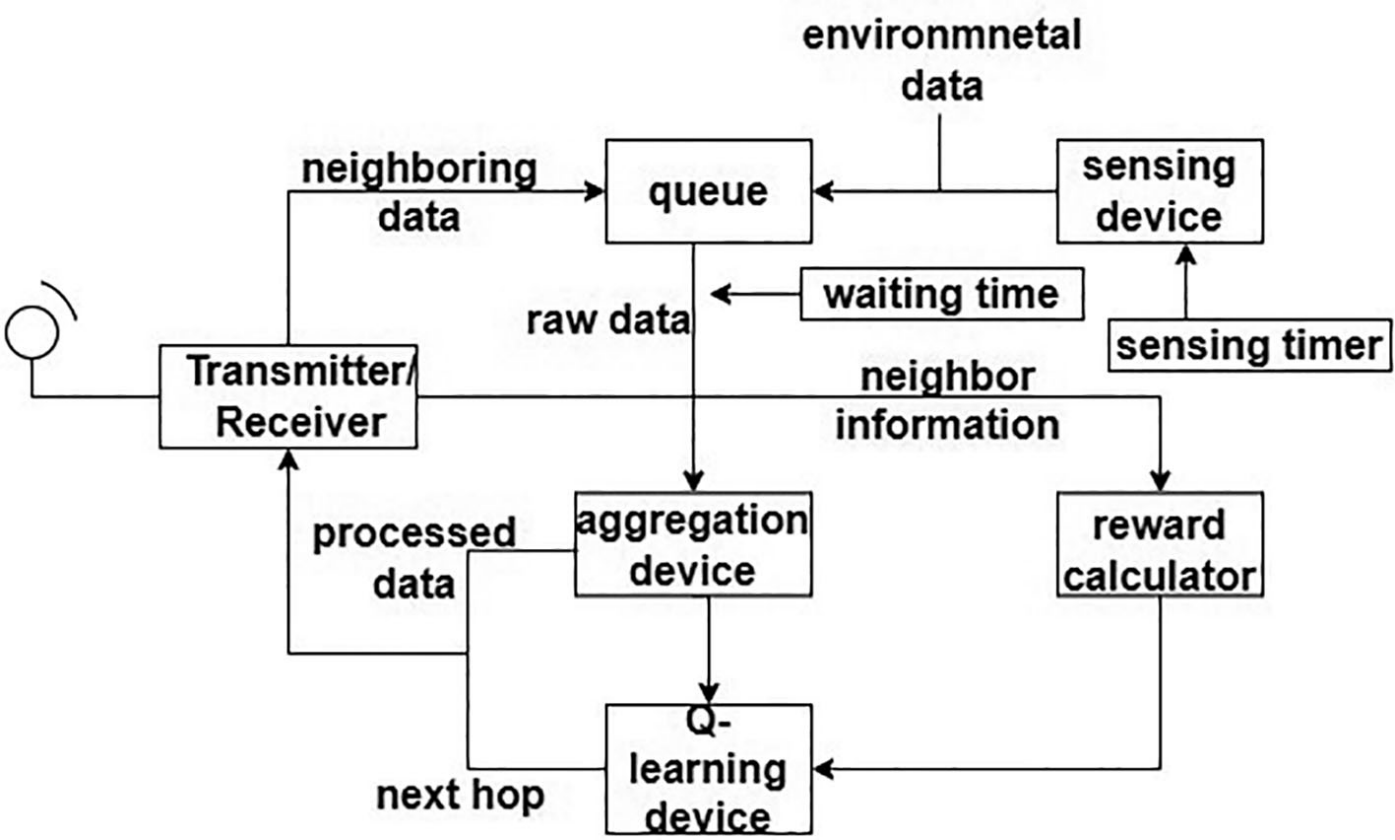
Schematic diagram of the method used. ^100^

Figure 6 shows the schematic for the system used in this paper. In order to locate a global best path, lower total energy consumption, and increase WSN lifespan, this work offers an energy-aware routing method. A node determines the potential levels of data aggregation from nearby nodes when it must select a routing path. Data from different sensor types (such as temperature and vibration measurement sensors) may not correlate strongly, therefore they cannot be combined. Utilizing the revised Q-values depending on the incentives, these SNs can select the best next node in the network in this way. This algorithm is compared with the “shortest path routing (SPR)” and “shortest path routing with data aggregation (SPRwDA)”.

Because the SPR and SPRwDA employ the shortest transmission path, which is only defined by the present network architecture, the energy usage at each time step is nearly constant. Since SPRwDA employs the suggested data aggregation technique prior to transmitting data at each node, it is clear that less energy is used than in SPR. The strategy dynamic reward update rule used in the proposed Q-DAEER technique causes each SN in the WSN utilizing this same proposed routing algorithm to have dynamic energy usage. Since data aggregation model 1 has the maximum efficiency, it uses the least energy on average compared to the other models. The suggested Q-DAEER can lower energy usage for 3 data aggregation models by 67%–32% compared to SPR and by 25%–5% compared to SPRwDA. The suggested Q-DAEER algorithm contains a data action selection algorithm and a Q-table updating technique, although the latter initially takes more time.

QSDN-WISE is a “QoS-based routing strategy that combines local network upkeep, a routing algorithm, and a clustering method” is proposed. ^101^ For software-defined WSNs, a brand-new QoS-based routing strategy based on SDN-WISE is suggested. A centralized architecture built on SDN-WISE is developed to allow complex network administration and boost system adaptability. It is advised to use a “double CH-based uneven clustering” (DCHUC) method to reduce the strain on CHs and avoid the energy hole problem. Built on SDN-WISE, QSDN-WISE is a QoS-based routing protocol that can support data with different QoS requirements. SDN-WISE framework, ^102^ “Disjoint multipath routing protocol” based on SDN (SDN-DMRP), ^103^ HEED, ^104^ “Improved Routing Protocol for Low power and Lossy networks” (IRPL), ^104^ and “Energy-efficient Unequal Clustering” (EEUC) are compared with the proposed protocol.

The network lives in suggested protocol and SDN-DMRP are greater than those in SDN-WISE under the assumption of the same number of nodes. As multi-path routing is used in SDN-DMRP, which distinguishes energy consumption, the NL is longer than in SDN-WISE. Due to clustering algorithm in QSDN-WISE takes into account the node remaining energy when selecting cluster-head nodes and employs the concept of non-uniform clustering to control the network energy consumption, the lifetime of the network in QSDN-WISE is significantly longer than in SDN-DMRP and SDN-WISE. In order to better balance the network’s energy usage, QSDN-WISE employs multi-path routing based on the double CHs and takes into consideration node residual energy.

“A new distributed mobile sink routing technique” is proposed. ^66^ In this study, a new hierarchical routing method for WSNs with a mobile sink called Ring Routing protocol is described. When using ring routing, a virtual ring structure is created that makes it simple to add new sink locations to the rings and for regular nodes to rapidly and effectively borrow sink locations from the rings as needed. The mobile sink selects the BS along sink’s path in order to relay sensor data to them. To avoid packet losses in the case that a SN’s sink position information becomes out-of-date, the sensor data is sent from the old BSs to the new BS. Ring Routing can be used with sensors which use asynchronously low-power MAC protocols designed for WSNs because it only necessitates a limited number of broadcasts. Ring routing just requires the MAC layer to be able to support broadcasts. It is suitable in situations when sink movement is unpredictable and will not depend on predicting the trajectory of the sink. Ring Routing’s performance is compared with “Line-Based Data Dissemination“(LBDD) ^105^ and Railroad ^106^ compared with the proposed protocol.

The typical energy usages of Ring Routing, LBDD, and Railroad for different sink speed values are compared. Ring routing provides optimum performance in every circumstance. Regarding values for sink speed, LBDD performs better than Railroad. There is a cost associated with the energy advantages of ring routing. In every scenario, Ring Routing results is better in terms of average energy consumption than LBDD and Railroad. Ring routing as well as LBDD both perform consistently across a range of network sizes, while Railroad’s performance suffers noticeably for bigger networks. By simply keeping the positions of each sink on the ring, it is simple to adapt the concept of Ring Routing to work with many mobile sinks. However, without considering the benefits of employing many sinks, this would merely be a change.

“A distributed robust routing protocol” is presented. ^79^ The contribution is the investigation of distributed EE stable routing for mobile WSNs. This suggested methodology only requires local knowledge because cooperative relay is carried out at each step. Lower layer coordination is required for multi-node cooperation. The dependable cooperative routing is centered on a cross-layer design that leverages the IEEE 802.11 scheduling scheme that has been proved to be efficient in past studies. After establishing a path between the source and destination nodes, robust cooperative routing may ensure delivery of packets both against temporary and permanent path breaking. Since more robust and dependable links are selected for routing, cooperation between nearby nodes also increased energy efficiency. Selecting dependable lines may lessen the need for retransmissions, conserving energy and cutting down on delays. Performance is significantly improved by the robust cooperative routing technique when mobility of nodes and connection error are present. “Destination Sequenced Distance Vector” (DSDV), ^107^ and “Adhoc on Demand Distance Vector” (AOMDV) ^108^ are compared here.

This efficient routing protocol uses more energy per bit as node mobility rises. Since control overhead experienced while path discovery does not change significantly with node mobility, the energy usage of AOMDV is only tangentially connected with node mobility. As anticipated, node mobility causes a dramatic increase in the energy usage of DSDV as frequent topology changes result in more overhead. Although at a far slower rate than DSDV, this resilient routing algorithm also requires more energy as mobility of nodes rises. Instead of creating a new E2E path, the best relay node with modest message exchange is chosen. When the maximal node mobility is low, RRP’s energy consumption is lower than AOMDV’s, but it gradually approaches it as the highest node mobility rises. The cause is that packets at high mobility must frequently go through the cooperative procedure. The freshly self-nominated node on the desired path also sends update messages more frequently to update path information, which explains the increase in energy usage. Due to the collaboration process and the longer back off delay, RRP transmits packets with a longer delay than DSDV.

“A new energy efficient routing strategy for 3D WSNs based on a delicate ant colony algorithm” proposed. ^109^ The causes behind the performance degradation of 2-D WSN routing protocol over 3-D WSNs is investigated.

**Figure 7.**
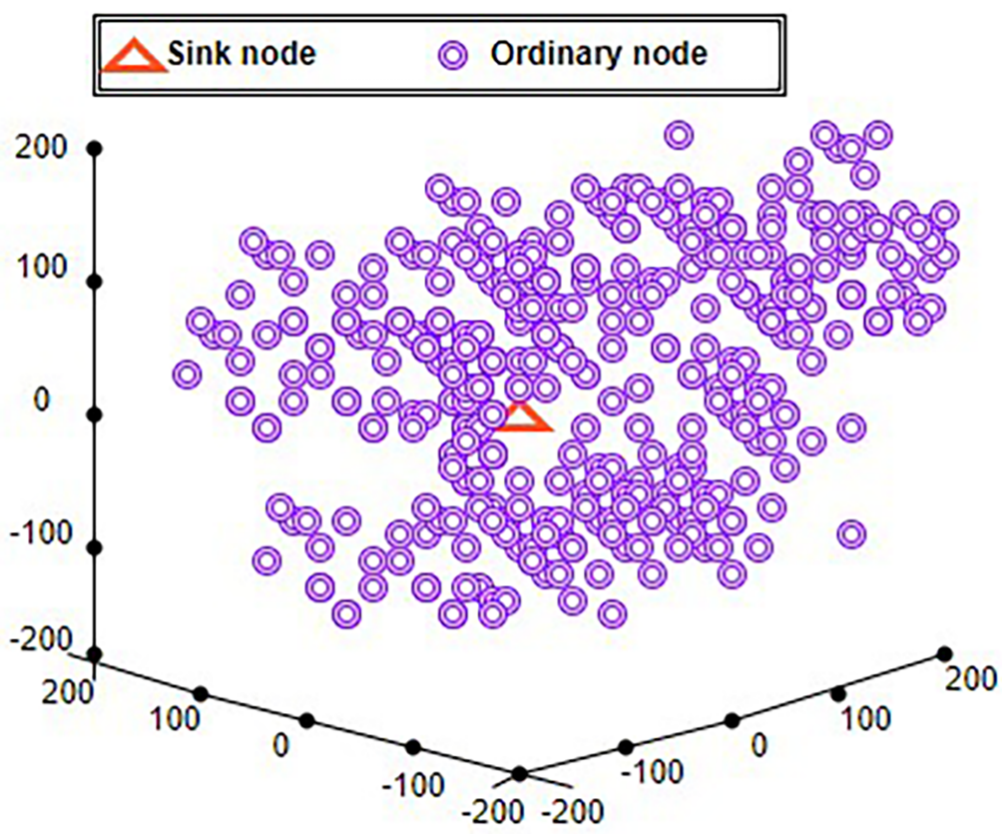
Sensor network model. ^109^

Figure 7 explains the proposed system model for the network. Sink node (SN) and ‘n’ regular nodes will be present in the network. The center of the sensor network is where the SN is located. Ordinary nodes (OD) cannot have their isomorphic energy raised and are randomly distributed in three dimensions. Every node has a unique ID on its own. The communication power of OD can be altered in accordance with the communication distance. CH nodes analyzes the information at the cluster nodes using the data fusion approach. Nodes regularly acquire data and continuously transfer data to SN. The nodes with lower overall distances from the nearby nodeshave a better probability of becoming CHs when the comparative distance among nodes and the neighboring nodes are taken into account. The SN assumes the OD role and utilizes the CH update mechanism to build a routing table unique to each CH. It is advised to use a novel path-finding method based on the ant colony method. The construction of wireless sensor networks is better suited for this technique. In addition to an overall length of the generated path, the algorithm also considers the energy of nodes that the path passes through. LEACH-3D, ^110^ “Advanced Zonal Stable Election Protocol” (AZ-SEP), ^111^ “Unequal clustering routing protocol” (UCNPD) ^112^ considering energy balancing based on “network partition and distance” are compared with the proposed protocol.

Even when the UCNPD-3D protocol, which is less successful than this protocol but still effective, loses half of its nodes, the average remaining energy of the nodes in this protocol is still higher than 50%. As a result, performance of the suggested algorithm is better than the other three protocols. This protocol utilizes the technique of SN route path generation and works in conjunction with the CH and SCH rotation mechanisms as well as the fragile collection ACO algorithm. This algorithm uses less energy throughout the clustering and route path construction processes, outperforming the other three protocols in terms of overall energy consumption.

Based on an “improved Archimedes optimization method, a WSNs routing” protocol (IAOAR) is proposed. ^113^ By improving the Archimedes optimization method, a fitness function is developed to identify the best CH location in relation to the distance between the virtual CH and the sink node as well as the energy is associated with the SN, which also controls energy usage between both the common node and the CH. The better position adjustments of CHs under the attraction of virtual force are researched in order to reduce the excessive energy loss caused by the near closeness of CHs. The augmented ACO algorithm is presented to find the minimal path among CHs for multi-hop data transfer. The improved ACO algorithm increases the ACO algorithms’ capability for global search by analyzing the ant scale, ant propagating direction, and changeover probability all through the entire search process. LEACH, “Energy consumption-based LEACH” (E-LEACH), ^114^ “Maximum-LEACH” (MAXLEACH) ^115^ are compared with the proposed protocol.

The protocol suggested in this study still has high energy compared to the other protocols that have virtually used up all of their energy. The primary explanation is that this algorithm, when choosing the CH, takes into account not just the node’s current energy but also its distance from the sink node and its location inside cluster. More crucially, the virtual force is employed to scatter the CHs equally as farther as possible, reduce transmission distances, and also consume less energy by repelling the CHs at close range. Even after the last remaining nodes of initial four algorithms have been exhausted, IAOAR nevertheless survives a significant number of nodes. This method contains fewer nodes during the same round than other algorithms. The reason is that this paper introduces multi-hop transmission along with balancing the consumption in the cluster. It uses an enhanced ACO algorithm to determine best route for transmitting information, which lowers the energy usage of CH nodes that are located far away from sink nodes and increases the network lifespan. However, the technique suggested in this research has to be further refined in heterogeneous WSNs whereas the effect of heterogeneous SNs on network’s energy usage is not taken into account.

“RowBee, a new routing technology built on the cross-technology communication (CTC)” principle is proposed. ^116^ To the best of our knowledge, the problem of a routing protocol based on CTC has never been studied in earlier literature. A simple but efficient routing method is described that use a WiFi gadget as a centralized coordinator to transmit the information to the neighboring ZigBee devices in order to reduce E2E delay. The ZigBee nodes will wakeup simultaneously in response to these beacons’ rendezvous in order to transmit data. The results of the investigation show how drastically RowBee’s simple architecture may reduce data transfer latency. Because it doesn’t require changing the node duty-cycle schedules, RowBee is very power-efficient.”Plant-Bioenergy MAC“(PB-MAC) ^117^ is compared with the new protocol.

This algorithm uses less energy than PB-MAC for just any number of nodes, and the energy consumption rises gradually as the number of sensors rises. Particularly, the energy expenditure disparity rapidly widens when more sensors are added to the situation. As the number of sensors nodes rises, so does the energy usage. However, RowBee consistently consumes less energy than PB-MAC.

WSN protocol for “secure and energy-conscious heuristic-based routing (SEHR)” is proposed. ^118^ The goal of this study was to suggest a WSN heuristic routing technique that is secure and energy-conscious.

**Figure 8.**
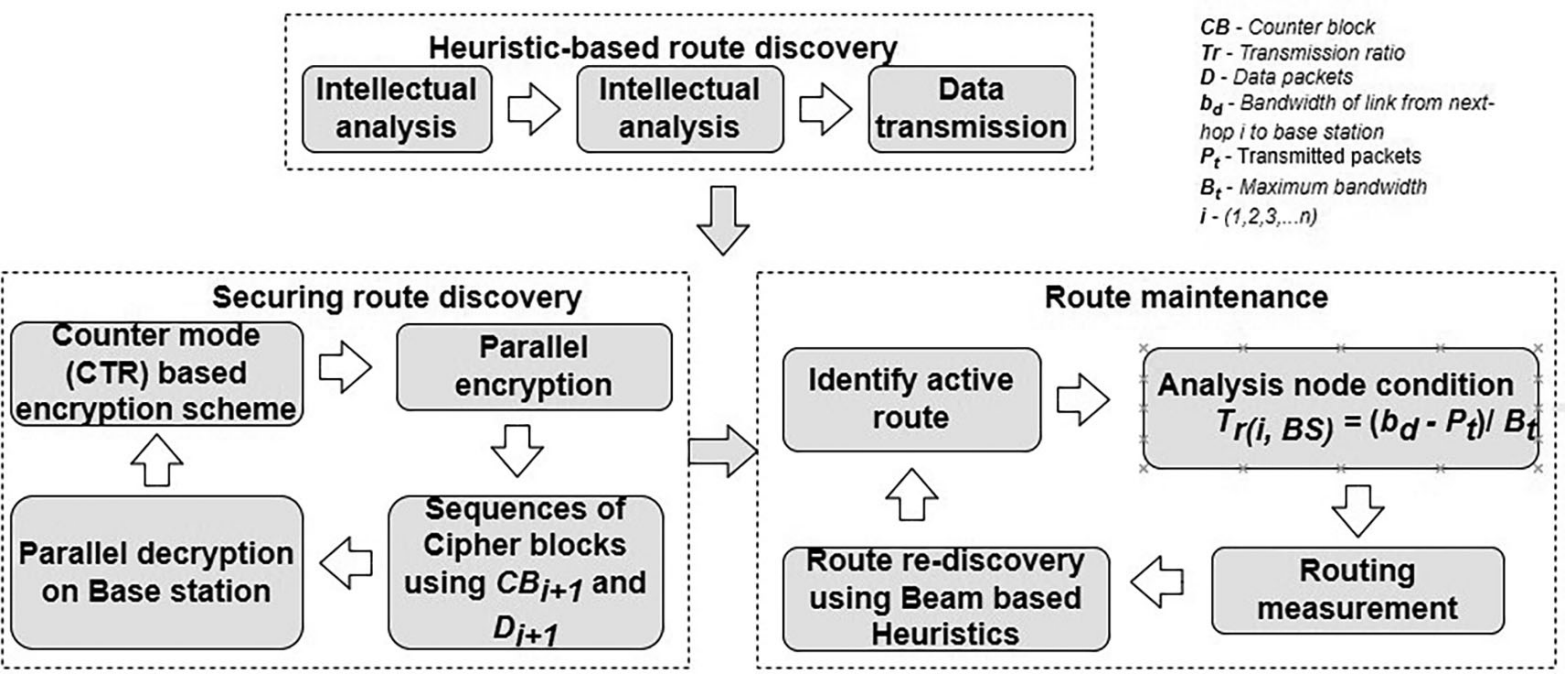
Architecture of secure and energy-conscious heuristic-based routing protocol. ^118^

Figure 8 depicts the architecture of SEHR protocol. It is classified into three modules as shown in the figure. Due of its limited resources, the SEHR protocol’s main objectives are effective utilization of energy, dependability, and secured information transfer performance. In this study, the beam heuristics-based SEHR protocol is used to achieve better data routing. It uses aggregate power, number of hop count, and integrity of link measurements to understand the decision of routing. The optimized results are produced by a beam-based heuristic algorithm, which was created in the recognized area of artificial intelligence. The processing and memory requirements on SNs are reduced by the proposed protocol, which also develops a graph-based solution is based on beam heuristics. The studied protocol also provides data authentication and encryption for inter-routing security using the cryptographic algorithm counter mode (CTR). Since the encryption of each datagram depends on the one before it, the proposed algorithm is more reliable and secure. The suggested work provides a method for route maintenance in addition to dynamic sensing and isolation of hostile nodes even during data security phase. The proposed protocol significantly enhances the network efficiency of low-powered sensor elements in terms of many network properties as compared to current cutting-edge alternatives. SecTrustRPL, ^119^ “heuristic-based EE” routing, ^120^ and PSO-based routing ^121^ are compared with the proposed protocol.

The SEHR protocol’s performance evaluation in comparison to the current solution is shown in terms of a range of node counts and data processing rates. In the presence of faults nodes, the SEHR protocol reduces energy consumption by an average of 39% and 33%, respectively. Due to its smart and fault-tolerant routing method, the SEHR protocol has been successful in stabilizing the routing direction for considerable amount of time. As a result, nodes with greater energy levels are chosen as data forwarders, minimizing the proportion of extra energy usage in route request and responder packets. The goal will be to enhance the SEHR protocol in the future by utilizing some simple machine learning-based approaches to provide the network more intelligence and fault tolerance.

“Sustainable multipath routing protocol” (SMRP) is proposed. ^122^ For multi-sink WSNs, a multi-path routing strategy called SMRP is recommended. To validate the effectiveness of proposed protocol in regards of endurance, efficiency, and delay of delivery, extensive simulation studies have been carried out. In SMRP, SN routing decisions are based on the local environment, the remaining energy, as well as the depth information. Two distributed multi-path routing algorithms are contrasted with SMRP: “Information Potential Field” (IPF) ^123^ and “Energy-aware dual-path geographical routing” (EDGR). ^77^

Proposed method performs best in three routing protocols in terms of PDR and “portion of living node” (PLN). Due to the fact that, EDGR and IPF will not take the external environment into account during routing, its pathways are susceptible to environmental influences. Transmission of data will become even more concentrated as more channels are closed for environmental reasons, which worsens the network’s energy imbalance. In contrast, SMRP can utilize the benefits of multi-path for energy balance more effectively because of high path chance of survival in challenging conditions. The efficiency of static multi-sink WSNs’ routing is main topic of this paper. Future work can expand proposed method to mobile multi-sink WSNs.

“Trust and energy aware routing protocol (TERP)” is proposed. ^124^ The shortcomings of current trust-based routing protocols have been specifically addressed by the development of a TERP. Given the resource - constrained nature of WSN, TERP was designed with dependability and energy efficiency in mind. TERP has the capacity to proactively identify and exclude bad nodes during the trust evaluation phase. The routing protocol, in contrast, features an energy awareness element during the route setup phase that enhances load balancing among trusted nodes. In addition to methods that ensure E2E paths are selected while taking the current levels of energy of the intermediate nodes under consideration, the TERP protocol was designed with trust-based routing integrated. This is essential because some network nodes’ high energy consumption can lead to their death and impair the network’s capacity to function. Since it is always desirable to choose short routes that need fewer wireless broadcast and cause fewer disturbance with the wireless medium, TERP also takes into account the total path length of any best path. Compared to other cutting-edge protocols like “Lightweight trust based routing protocol “(LTB-AODV)”, ^125^ and “Trust-aware secure routing framework” (TSRF), ^126^ the TERP is briefed as follows.

Since TERP’s architecture emphasizes reliability and energy efficiency, it keeps a significantly excellence in terms of computational time since the composite routing metric chooses reliable and EE nodes for routing. When nodes are chosen based on trust parameters, both LTB-AODV and TSRF don’t carry out any load balancing. Instead, they stay a part of effective routes until their energy runs out. As a result, existing systems perform inadequately in terms on network longevity under conditions of high network traffic. The TERP scheme provides a simple solution for SNs with limited resources, but its effectiveness on actual hardware platforms has to also be verified. Future work might be thought of as a modest testbed for the actual implementation of the suggested TERP method.

“An energy and temperature-aware, weighted, QoS-based routing protocol” known as (WETRP) is proposed by Ref. 127. The two steps of the suggested method are route identification and route maintenance. Hotspots and energy-poor nodes are avoided during route discovery which determines the shortest path with the lesser amount of link lag among both the sender and the receiver node. The “thermal-aware routing algorithm” (TARA), ^128^ and “Hotspot preventing routing” (HPR) ^129^ are compared with the proposed work.

The network resource determines a network’s lifespan. The lifespan of network is increased *via* better resource management. In terms of network longevity, WETRP beats other systems because TARA and HPR concentrate on eliminating hotspot nodes and locating the coolest neighbors, respectively. This causes inefficient use of valuable resources, which causes network nodes to lose energy more quickly. WETRP, on the other hand, is thermal and energy-aware strategy that maximizes NLby making an intelligent choice about temperature and energy, giving both variables equal weight. Future versions of this study could be tailored to address the problems brought on by postural body movement. Additionally, the suggested approach can incorporate packet-level priority so that important data packets can be distinguished from non-critical data packets and routed according to priority.

## Comparison of enhanced energy efficient protocols reviewed

The various EE protocols are listed in Table 2. Their comparison with the factors of energy efficiency are listed which could help researchers and academicians to understand the research gaps in WSNs. It help to understand the factors to improve lifetime of WSNs.

**Table 2.** Comparison of enhanced energy efficient protocols in wireless sensor networks.

Reference Number	Enhanced energy efficient protocol	Compared protocol	Type of protocol	Factors considered for comparison	Merits	Demerits
^25^	“3-Dimensional real-time geographic routing”	“Above below location aided routing” and 3D Greedy routing	Geographical routing	End to end packet delay, Packet miss ratio	Lesser energy consumption	Not applicable for mobile SNs
^27^	“General self-organized tree-based energy-balance routing protocol”	“Low energy adaptive clustering hierarchy”, “Hybrid energy efficient distributed”, “Power efficient gathering in sensor information systems”, “Power efficient data gathering and aggregation protocol”, “Tree based energy efficient protocol for sensor information” and “Tree based clustering”	Hierarchical (tree-based)	Network lifetime	More efficient and longer network lifetime	Increases transmission delay
^34^	“Fault-tolerant clustering routing algorithm established on a gaussian network for wireless sensor networks	“Fault tolerance-LEACH”, “Hybrid energy efficient distributed” and “Particle swarm optimization based unequal fault tolerance clustering”	Hierarchical	Energy efficiency and dead nodes, Data reliability	High data reliability and energy efficiency of nodes	Difficult to connect long distance nodes, which in turn increases latency
^36^	“Robust and em multicast routing”	“Hierarchical geographical multicast routing”, “Robust and scalable geographic multicast” and “Multicast routing with branch information nodes”	Geographic multicast routing	Sum of path length, Multi-level facility, Packet-loss recovery	Higher node density has lower packet loss	Path sum length increases without using “Trajectory-based Lightweight Hole Detection”
^39^	“Range-based opportunistic routing”	“Energy efficient opportunistic routing” and“Extremely opportunistic routing”	Opportunistic routing	Energy consumption, Packet drop ratio	Better packet drop ratio	Implementation is difficult for a vast network
^43^	“Effective bypassing void routing protocol, which is based on a virtual routing circle”	“Greedy perimeter stateless routing”	Hierarchical geographical based	Average delivery ratio, Transmission delay, Average hops	Minimized average hop in turn reducing average delay	Packet delivery ratio is less
^45^	“An efficient improved artificial bee colony algorithm”	“LEACH-Centralized”, “Fitness value based improved grey wolf optimization” and “artificial bee colony- SD"	Hierarchical	Lifetime, Total residual energy, Stability period, Throughput	Energy balance of nodes is very well maintained	Only implemented for stationary node network
^49^	“Dynamic cluster-Based static routing protocol”	“Dynamic duty cycle”, “Energy Efficient connected coverage scheme” and “Optimal clustering in circular networks	Hierarchical	Communication cost, Computation cost, Energy consumption, Throughput.	Network load is managed evenly	Initial implementation of this model is difficult
^53^	3D cluster protocol	Main-3D, Leach-3D	3D-hierarchical	Sensor Nodes which are active with respect to time, Throughput, Energy consumption, Lifetime	signal to noise ratio rises makes the channel quality requirement rise.	Network lifetime reduces due to data retransmission
^56^	“Distributed 2-hop cluster routing protocol”	“Low energy adaptive clustering hierarchy”, “Power efficient gathering in sensor information systems”, “Residual energy based - LEACH" and “Two tier distributed fuzzy logic-based Protocol”	Hierarchical cluster-based	Optimal Number of 2-hop clusters, Network lifetime, Energy consumption, Raw packets to Basestation, Scalability	Using 2 hop clusters, improves energy efficiency	Not tested for mobile network
^60^	Virtual-infrastructure-based “Delay-aware green routing protocol”	“Ring routing protocol” and “Grid-cycle routing protocol”	Hierarchical area based	Number of Sensor Nodes, Sink speed, Network size	Building multiple rings helps in better energy utilization	Data delivery for mobility nodes has to considered
^63^	“Destination oriented routing algorithm”	“Power efficient gathering in sensor information systems”, and “random projection-polar coordinate-chain routing”	Hierarchical	Network lifespan, Packet transmission	Network lifespan is much better	Evaluating the polar angle and transmitting radius is difficult
^65^	“Energy efficient heterogeneous ring clustering”	“Routing protocol for low-power and lossy networks”	Hierarchical Multipath Based Routing	Energy consumption, Packet loss ratio, Packet Delivery Ratio	Cluster Head selection can be easily done	Fluctuation range of node energy in Wireless sensor network is higher
^66^	“Energy efficient and reliable routing protocol for mobile wireless sensor networks”	“Mobile - low energy adaptive clustering hierarchy"	Hierarchical	Throughput, Network lifetime	Packet delivery is maintained even despite node movement	As the network area rises average energy also rises
^68^	“An efficient and secure routing protocol for wireless sensor networks through single to noise ratio-based dynamic”	“Low energy adaptive clustering hierarchy” and “Power efficient gathering in sensor information systems”	Hierarchical	Packet Delivery Ratio, End to end delay	Due to better clustering, node power is managed	Not used for homogeneous networks
^69^	“Biologically inspired secure autonomous routing protocol”	“Energy and delay model based on ant algorithms”, “Secure real-time load distribution” and “Improved energy efficient ant based routing”	Meta-heuristic algorithms (Geo-directional protocol)	Routing Load, Energy consumption	Due to proper analysis of data packets, routing is done efficiently	Using multiple algorithm causes difficulty in implementation
^72^	“Efficient dual-path geographical routing”	“Energy-efficient beaconless geographic routing”, “Energy-efficient multicast geographical routing”, and “Power adjusted greedy- cordinate face(1)”	Geographic protocol	Energy consumption, Lifetime of network, Packet Delivery Ratio, Delivery latency	Use of two anchors effectively prevents data going beyond the cluster boundary	When communication overload occurs, consumes more energy
^75^	“Enhanced balanced energy-efficient network-integrated super-heterogeneous”	“Stable election protocol”, “Distributed energy efficient clustering" and “Centralized energy efficient clustering”	Hierarchical	Network lifecycle, Residual energy	Provides even energy distribution of network and also increases lifetime	Network jitter is not considered
^79^	“Multi-threshold segmentation-based energy efficient routing”	“Residual energy based - LEACH", “Fitness value based improved grey wolf optimization” and “Clustering routing protocol based on fuzzy C means”	Hierarchical	Residual energy, Network lifetime	Energy is consistent throughout the network	Variable load factor on Cluster Heads is not considered
^80^	“Energy-saving clustering by voronoi adaptive dividing”	“Minimum transmission energy”, “Low energy adaptive clustering hierarchy”, “Centralized-LEACH”, “Stable election protocol” and “Threshold sensitive energy efficient sensor network”	Hierarchical cluster-based	Network lifetime, Energy consumption	High residual energy	Mobility of sink node should be evaluated
^85^	“Multi-hop routing protocol built on game theory and coverage optimization”	“Localized game theoretical clustering algorithm”, “Residual energy based LEACH” and “Energy efficient clustering algorithm based on game theory”	Hierarchical cluster-based	Energy consumption, Network lifetime	Using same energy more data packets can be transferred	Node death rounds has to be further improvised
^88^	“Energy and traffic aware sleep-awake"	“Traffic and energy aware routing” and “Sleep-awake energy efficient distributed”	Hierarchical cluster-based	Network lifetime, Throughput, Residual energy	Load balance is enhanced by the Cluster Head selection process	Traffic variability of network is to be examined
^91^	“Mobile sink-based adaptive immune energy efficient clustering protocol”	“Low energy adaptive clustering hierarchy” and “Mobile sink improved energy efficient PEGASIS-based”	Hierarchical	Network lifetime, Stability period, Instability period, First dead node, Half dead node, Last dead node Alive nodes per round, Throughput, Packet drop ratio,	Mobility of sink excels the network’s life and throughput	Stability period of mobile nodes is more
^94^	“Mode-switched grid-based routing protocol for wireless sensor network”	“Energy aware grid based routing”	Hierarchical grid based	Network lifetime, Packet drop ratio, End to end delay, Average energy consumption	Flooding of control packets is reduced	More mobile nodes have to be incorporated
^93^	“Straight line routing”	“Roumor routing”	Flat networks routing protocols	Path discovery ratio, Energy consumption, Hop stretch	Less energy cost	Only efficient for small networks
^95^	“Q-learning-based data aggregation-awareenergy efficient routing protocol”	“Shortest path routing" and “Shortest path routing with data aggregation”	Hierarchical cluster-based	Energy consumption, Network lifetime, Average hop count	Efficient data aggregation model	Q-table updating takes more time
^96^	“QoS-based routing strategy based on SDN-WISE"	“Software defined networks for wireless sensor networks framework”, “Software defined networks – disjoint multipoint routing protocol”, “Hybrid energy efficient distributed”, “Improved routing protocol for low power and lossy networks” and “Energy-efficient unequal clustering”	QoS-based routing protocol with clustering and routing algorithm	Control message overhead, Packet loss rate, End to end delay, Network lifetime	Good network lifetime	Less nodes are considered
^64^	“A novel, distributed, energy-efficient mobile sink routing protocol”	“Line-based data dissemination” and Railroad	Hierarchical area based	Delay breakdown, Network lifetime, Energy consumption	Average energy consumption is better	More sinks has to be established
^74^	“A distributed robust routing protocol”	“Destination sequenced distance vector” and “Adhoc on demand distance vector”	Cross layer robust routing	Packet Delivery Ratio, End to end delay, Energy consumption	Low node mobility provides lesser energy consumption	Packet transmission delay is more
^104^	“Advanced energy optimized routingbased on a fragile ant colony optimization in 3D wireless sensor networks”	“Low energy adaptive clustering hierarchy-3D”, “Advanced zonal stable election protocol” and “Unequal clustering routing protocol considering energy balancing based on Network partition and distance”	Hierarchical	Energy consumption, Load balance of the network, Network lifetime	During clustering and routing path construction less energy is consumed	Cannot be applicable for more number of network nodes
^105^	Improvised archimedes optimization method, a wireless sensor networks routing protocol	“Low energy adaptive clustering hierarchy”, “Energy consumption based low energy adaptive clustering hierarchy” and “Max low energy adaptive clustering hierarchy”	Hierarchical	Residual energy, Network lifetime, Energy consumption	Cluster Heads not only considers the energy of nodes but also the distance	Not applicable for heterogeneous network
^108^	“A new routing protocol based on a CTC technique called RowBee”	“Plant-Bioenergy MAC”	Cross technology communication based	End to end delay, Energy consumption	Use of Wi-Fi gadgets coordinates the data transmission	As the nodes increases energy usage also increases
^110^	“Secure and energy-conscious heuristic-based routing”	SecTrustRPL, Heuristic-based EE routing, PSO-based routing	Heuristic-based	Network lifetime, Energy consumption, Throughput, Packet drop ratio, Faulty routes, Network overhead	Reliable and secure because of data encryption	More machine learning and fault tolerance algorithm has to be used for more efficiency
^114^	“Sustainable multipath routing protocol”	“Information potential field” and “Energy-aware dual-path geographic routing”	multipath routing protocols	Network lifetime, Portion of living nodes, Packet Delivery Ratio, End to end Delay, Energy balance factor	Using multi-path channels helps energy consumption	Node mobility has not be considered
^116^	“Trust and energy aware routing protocol”	“Lightweight trust based routing protocol” and “Trust-aware secure routing framework”	Trust-based	Throughput, End to end delay, Routing load, Network lifetime	Using load balancing, it provides better efficiency and network longevity	Used for Sensor Nodes with limited resources
^119^	“An energy and temperature-aware, weighted, QoS-based routing protocol”	“Thermal-aware routing algorithm” and “Hotspot preventing routing”	QoS-based	Packet Delivery Ratio, End to end delay, Throughput, Temperature rise, Network lifetime, Normalized routing load	Thermal and energy-aware strategies enhance the network lifetime	Critical and non-critical data packets are not distinguished

## Issues and challenges of energy-efficient protocols in WSNs

WSN protocols must overcome many challenges so that it can be more EE and more precise. One of major factor which must be analyzed in WSNs is the mobility of nodes in the network which must be considered while designing a protocol. When the distance between the nodes in WSN is more, packet delay and sum of path length will be increased which further affects the efficiency of the network. Hence an efficient protocol should take care of this parameter. In some protocols the implementation will be very complex which will not be very effective for real time applications. The protocols must be optimized for both heterogeneous WSNs and energy consumption. The heterogeneous WSN’s lifespan can be improved, but care must be taken to reduce the complexity of the protocols. WSNs which involve large areas of network coverage, the protocols should be able to consider multiple clusters and CH selection becomes the most important factor which will help in reducing the energy utilization of nodes. The protocols should consider congestion and also data transmission due to clustering and multiple hops among the clusters. Asynchronous duty cycles between the SNs can also help to increase energy economy and routing performance.

## Conclusions

In real life scenario, there are widespread applications of WSNs. The integration of the IoT with WSNs has been of great interest in recent times. This makes the WSN technology an open area for advancement in research and development. In the past years, more involvement is shown by a lot of researchers/academicians across the globe in this area where works have been carried out to make the WSNs more EE, improving the lifespan of the networks. Based on the above factors, in this work a sincere attempt is made to compare different protocols which make the WSNs more efficient with respect to utilization of energy, lifespan of the network, throughput and so on. Different protocols have been considered in this work, which are thoroughly compared with the standard protocols based on various factors which makes the protocols considered in this review more EE than the already existing ones. Apart from the various factors which have been considered in this article for making the WSNs more EE, there are many more parameters which can be still taken up or future study. This limitation can be overcome by researchers by studying the parameters not considered in this article. The work will help as a one stop check point for the researchers as it gives more insight into the factors that make the protocols understudy more EE and robust.

## Data Availability

No data are associated with this article.
