# Supplementary material for: A comprehensive review of energy efficient routing protocols for query driven wireless sensor networks
Source: F1000Res. 2024 Jun 4;12:644. Originally published 2023 Jun 12. [Version 3] doi: 10.12688/f1000research.133874.3 (PMC11157196; doi:10.12688/f1000research.133874.3)
Supplement: Peer review history

# Reviewer report: Reviewer response for version 3

**DOI:** 10.5256/f1000research.167296.r286139

**Published:** 2024-06-06

**Authors:** Michaelraj Kingston Roberts

**Affiliations:** 1 Sri Eshwar College of Engineering, Coimbatore, India

All my queries have been addressed. This work can now be accepted.

Is the review written in accessible language?

Yes

Are all factual statements correct and adequately supported by citations?

Yes

Are the conclusions drawn appropriate in the context of the current research literature?

Partly

Is the topic of the review discussed comprehensively in the context of the current literature?

Partly

Reviewer Expertise:

WSN, Error Control Coding

I confirm that I have read this submission and believe that I have an appropriate level of expertise to confirm that it is of an acceptable scientific standard.

# Reviewer report: Reviewer response for version 2

**DOI:** 10.5256/f1000research.164550.r274810

**Published:** 2024-05-24

**Authors:** Michaelraj Kingston Roberts

**Affiliations:** 1 Sri Eshwar College of Engineering, Coimbatore, India

While the article comments on issues and challenges, expanding this discussion to include potential solutions or ongoing research efforts that address these challenges could greatly enhance the practical relevance of the review. Additionally, discussing the trade-offs between energy efficiency and other performance metrics like latency or reliability could provide a more balanced view.

 Ensure that the literature review includes the most recent and relevant studies. Given the rapid advancement in wireless sensor networks and IoT, it is crucial that the review reflects the latest trends and developments. This will make the article more valuable for researchers and practitioners who are looking for the most current information. The following research works shall be included to enhance the overall scope of the current problem statement:

 1. Roberts M.et.al., 2022 (Ref 1)

 2.  Priyadarshi R.et., 2024 (Ref 2)

 3. Liu Y.et.al., 2023 (Ref 3)

 4. Surenther I.et.al., 2023 (Ref 4)

Is the review written in accessible language?

Yes

Are all factual statements correct and adequately supported by citations?

Yes

Are the conclusions drawn appropriate in the context of the current research literature?

Partly

Is the topic of the review discussed comprehensively in the context of the current literature?

Partly

Reviewer Expertise:

WSN, Error Control Coding

I confirm that I have read this submission and believe that I have an appropriate level of expertise to confirm that it is of an acceptable scientific standard, however I have significant reservations, as outlined above.

## References

[1] : An optimized ticket manager based energy‐aware multipath routing protocol design forIoT based wireless sensor networks. *Concurrency and Computation: Practice and Experience* .2022;34(28) : 10.1002/cpe.7398 10.1002/cpe.7398

[2] : Energy-Efficient Routing in Wireless Sensor Networks: A Meta-heuristic and Artificial Intelligence-based Approach: A Comprehensive Review. *Archives of Computational Methods in Engineering* .2024;31(4) : 10.1007/s11831-023-10039-6 2109-2137 10.1007/s11831-023-10039-6

[3] : DCC-IACJS: A novel bio-inspired duty cycle-based clustering approach for energy-efficient wireless sensor networks. *Journal of King Saud University - Computer and Information Sciences* .2023;35(2) : 10.1016/j.jksuci.2023.01.015 775-790 10.1016/j.jksuci.2023.01.015

[4] : Maximizing energy efficiency in wireless sensor networks for data transmission: A Deep Learning-Based Grouping Model approach. *Alexandria Engineering Journal* .2023;83: 10.1016/j.aej.2023.10.016 53-65 10.1016/j.aej.2023.10.016

# Reviewer report: Reviewer response for version 2

**DOI:** 10.5256/f1000research.164550.r266871

**Published:** 2024-04-18

**Authors:** Sarang Karim

**Affiliations:** 1 Quaid-e-Awam University of Engineering, Science and Technology, Nawabshah, Pakistan

I am happy that the authors have included all my recommendations and suggestions, Paper can be accepted now.

Is the review written in accessible language?

Yes

Are all factual statements correct and adequately supported by citations?

Partly

Are the conclusions drawn appropriate in the context of the current research literature?

Partly

Is the topic of the review discussed comprehensively in the context of the current literature?

Partly

Reviewer Expertise:

IoT, WSN, UWSN, Routing Protocols, Smart Agriculture, Machine Learning, Water Quality Analysis

I confirm that I have read this submission and believe that I have an appropriate level of expertise to confirm that it is of an acceptable scientific standard.

# Reviewer report: Reviewer response for version 1

**DOI:** 10.5256/f1000research.146889.r226763

**Published:** 2024-02-14

**Authors:** Sarang Karim

**Affiliations:** 1 Quaid-e-Awam University of Engineering, Science and Technology, Nawabshah, Pakistan

The authors conduct a comprehensive survey on energy-efficient WSN routing protocols; however, there are major flaws in the article:

 The work is updated and currently focused by the many researchers around the globe. So I validated their work, but the work must be comprehensive and compared with the latest existing survey papers

 There are many survey articles on WSN; authors must compare their surveys with existing surveys in terms of table and descriptions.

 The authors suggest future analysis to make WSN more energy-efficient without specifying how this analysis will be conducted or the expected outcomes. Providing more clarity on the proposed future work would strengthen the context.

 The section "Issues and challenges of energy-efficient protocols in WSNs" needs more explanation and justification.

 The section "Issues and challenges of energy-efficient protocols in WSNs" must also proposed new trends and solutions to tackle the challenges.

 Authors are encouraged to cite the latest papers from 2023.

Is the review written in accessible language?

Yes

Are all factual statements correct and adequately supported by citations?

Partly

Are the conclusions drawn appropriate in the context of the current research literature?

Partly

Is the topic of the review discussed comprehensively in the context of the current literature?

Partly

Reviewer Expertise:

IoT, WSN, UWSN, Routing Protocols, Smart Agriculture, Machine Learning, Water Quality Analysis

I confirm that I have read this submission and believe that I have an appropriate level of expertise to state that I do not consider it to be of an acceptable scientific standard, for reasons outlined above.

# Response

**Published:** 2024-04-02

**Authors:** Pramod Kumar

**Affiliations:** Electronics & Communication, Manipal Institute of Technology, Manipal, Karnataka, India

We have updated article as per reviewer comments and incorporated all the changes in the article.
